# Microbiome responses during virulence adaptation by a phloem‐feeding insect to resistant near‐isogenic rice lines

**DOI:** 10.1002/ece3.5699

**Published:** 2019-10-04

**Authors:** Finbarr G. Horgan, Thanga Suja Srinivasan, Eduardo Crisol‐Martínez, Maria Liberty P. Almazan, Angelee Fame Ramal, Ricardo Oliva, Ian L. Quibod, Carmencita C. Bernal

**Affiliations:** ^1^ EcoLaVerna Integral Restoration Ecology Kildinan Ireland; ^2^ University of Technology Sydney Sydney NSW Australia; ^3^ Centre for Plant Molecular Biology and Biotechnology Tamil Nadu Agricultural University Coimbatore India; ^4^ International Rice Research Institute Metro Manila Philippines; ^5^ Centre for Climate Change Studies Sathyabama Institute of Science and Technology Chennai India; ^6^ COEXPHAL (Association of Vegetable and Fruit Growers of Almeria) Almeria Spain; ^7^ School of Environmental Science and Management University of the Philippines Los Baños Philippines

**Keywords:** detoxification, host plant resistance, mutualism, *Nilaparvata lugens*, virulence adaptation, yeast‐like symbionts

## Abstract

The microbiomes of phloem‐feeding insects include functional bacteria and yeasts essential for herbivore survival and development. Changes in microbiome composition are implicated in virulence adaptation by herbivores to host plant species or host populations (including crop varieties). We examined patterns in adaptation by the green leafhopper, *Nephotettix virescens*, to near‐isogenic rice lines (NILs) with one or two resistance genes and the recurrent parent T65, without resistance genes. Only the line with two resistance genes was effective in reducing leafhopper fitness. After 20 generations on the resistant line, selected leafhoppers attained similar survival, weight gain, and egg laying to leafhoppers that were continually reared on the susceptible recurrent parent, indicating that they had adapted to the resistant host. By sequencing the 16s rRNA gene, we described the microbiome of leafhoppers from colonies associated with five collection sites, and continually reared or switched between NILs. The microbiomes included 69–119 OTUs of which 44 occurred in ≥90% of samples. Of these, 14 OTUs were assigned to the obligate symbiont *Candidatus sulcia* clade. After 20 generations of selection, collection site had a greater effect than host plant on microbiome composition. Six bacteria genera, including *C. sulcia*, were associated with leafhopper virulence. However, there was significant within‐treatment, site‐related variability in the prevalence of these taxa such that the mechanisms underlying their association with virulence remain to be determined. Our results imply that these taxa are associated with leafhopper nutrition. Ours is the first study to describe microbiome diversity and composition in rice leafhoppers. We discuss our results in light of the multiple functions of herbivore microbiomes during virulence adaptation in insect herbivores.

## Graphical abstract

We describe the microbiome of leafhoppers from colonies associated with five collection sites and continually reared, or switched between near‐isogenic rice lines with resistance genes. The microbiomes included 69–119 OTUs of which 44 occurred in ≥90% of samples. Six bacteria genera, including *Candidatus sulcia*, were associated with leafhopper virulence. Ours is the first study to describe microbiome diversity and composition in rice leafhoppers.

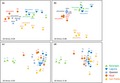

## 1 INTRODUCTION

The phloem of grasses is consumed by a diversity of specialized insect herbivores. Although rich in sugars and nutrients, phloem is deficient in several amino acids that are the necessary building blocks of insect proteins (Gündüz & Douglas, 2009; Wan et al., 2014). Furthermore, grasses have evolved a range of defenses to deter herbivores and to reduce the fitness gains from phloem feeding. Many of these defenses are located in the phloem, including secondary chemicals and herbivore‐induced defense hormones such as jasmonic (JA) and salicylic acids (SA) or enzyme‐blocking molecules such as proteinase inhibitors (Behmer et al., 2013; Du et al., 2015; Fujita, Kohli, & Horgan, 2013). It is now apparent that phloem‐feeding insects depend on a range of facultative or obligate symbiotic microorganisms, including bacteria and fungi, to overcome the nutritional deficiencies and defenses of phloem (Douglas, 1998; 2009; Ferrater, Jong, Dicke, Chen, & Horgan, 2013; Hansen & Moran, 2014; Noda et al., 2012; Sasaki, Kawamura, & Ishikawa, 1996; Wan et al., 2014). Furthermore, a number of studies suggest that endosymbiotic bacteria and yeasts might determine host preferences among phloem feeders (Ferrari, Scarborough, & Godfray, 2007; Ferrari, West, Via, & Godfray, 2012; Ferrater et al., 2015). Moreover, based on evidence from manipulative studies with plant bugs (*Megacopta* spp.), Hosokawa, Kikuchi, Shimada, and Fukatsu (2007) indicated that symbionts can determine the comparative virulence of bug species on different host plants. Several authors have proposed that changes in symbiotic bacterial and yeast communities may also underlie the rapid adaptation by pest herbivores to resistant crop varieties (Chen, Bernal, Tan, Horgan, & Fitzgerald, 2011; Ferrater & Horgan, 2016; Ferrater et al., 2015; Lu et al., 2004; Tang, Lv, Jing, Zhu, & He, 2010; Wang, Zhu, Lai, & Fu, 2015; Xu et al., 2015). Knowledge of the role of the microbiome is therefore essential to fully understand the ability of herbivores to overcome host defenses—a major component of insect–plant coevolution (Stenseth & Smith, 1984).

Over the last 60 years, host plant resistance has received considerable research attention in the management of cereal pests, particularly phloem‐feeding hemipterans. In Asia, plant resistance against rice planthoppers and leafhoppers is currently the principal focus of publically funded research into insect pest management (Horgan, 2018). However, the widespread and rapid adaptation by target herbivores to resistant hosts is a major challenge for crop breeders and seriously limits the effectiveness and durability of resistant crop varieties (e.g., aphids on wheat, planthoppers, and leafhoppers on rice [Haley, Peairs, Walker, Rudolph, & Randolph, 2004; Hirae, Fukuta, Tamura, & Oya, 2007; Horgan, 2018; Horgan et al., 2015, 2018; Vu et al., 2014]). Recent studies that examined the microbiomes of insect herbivores, particularly the rice planthopper, *Nilaparvata lugens*, reared‐on or adapted to contrasting host plant genotypes have suggested that microbiomes change (structurally or functionally) during selection and possibly determine adaptation (Ferrater et al., 2013, 2015; Ojha, Sinha, Padmakumari, Bentur, & Nair, 2017; Wang et al., 2015). However, Ferrater et al. (2013) indicated that to date, most studies that seek associations between endosymbionts and herbivore virulence have only described microbiomes from two or three insect colonies or samples, each reared on a different host plant (e.g., Chen et al., 2011; Lu et al., 2004; Ojha et al., 2017; Tang et al., 2010; Xu et al., 2015). This design confounds microbiome variability between different herbivore populations and microbiome responses to host feeding. Furthermore, until recently, virulence studies of rice planthopper microbiomes have used rice varieties from diverse breeding backgrounds often without clear knowledge of the genetic mechanisms underlying resistance. For example, the varieties TN1 (susceptible), Mudgo (*Bph1* locus), and ASD7 (*bph2* locus) have been used in several studies of rice planthopper microbiomes despite widespread virulence of planthoppers to all three varieties (Chen et al., 2011; Wang et al., 2015; Xu et al., 2015). These factors make it difficult to relate changes in microbiomes to the selection potential of specific resistance genes. Recently, a number of research teams have developed near‐isogenic rice lines that share common recurrent parents, but differ by containing specific gene loci introgressed through marker‐assisted selection from different resistance donors (Fujita et al., 2013; and see Horgan et al., 2019). By using such near‐isogenic lines, studies of virulence adaptation can better associate changes in microbiomes with the effects of specific resistance genes without confounding background genetic effects (Horgan, 2018).

Leafhoppers (Hemiptera: Cicadellidae) form symbioses with a range of bacteria, including obligate endosymbionts that occupy specialized bacteriomes in the insect's anterior abdomen (Noda et al., 2012). Although a number of endosymbiotic bacteria have been identified from rice leafhoppers (e.g., *Candidatus sulcia* and *Candidatus nasuia* clades, *Rickettsia* sp., and *Wolbachia* sp., Kittayapong, Jamnongluk, Thipaksorn, Milne, & Sindhusake, 2003; Noda et al., 2012; Watanabe, Yukuhiro, Matsuura, Fukatsu, & Noda, 2014), to our knowledge no previous study has used amplicon sequencing (i.e., targeted gene sequencing) to describe the microbiome of rice leafhoppers or to examine changes in the leafhopper microbiome during virulence adaptation.

**Figure 1:**
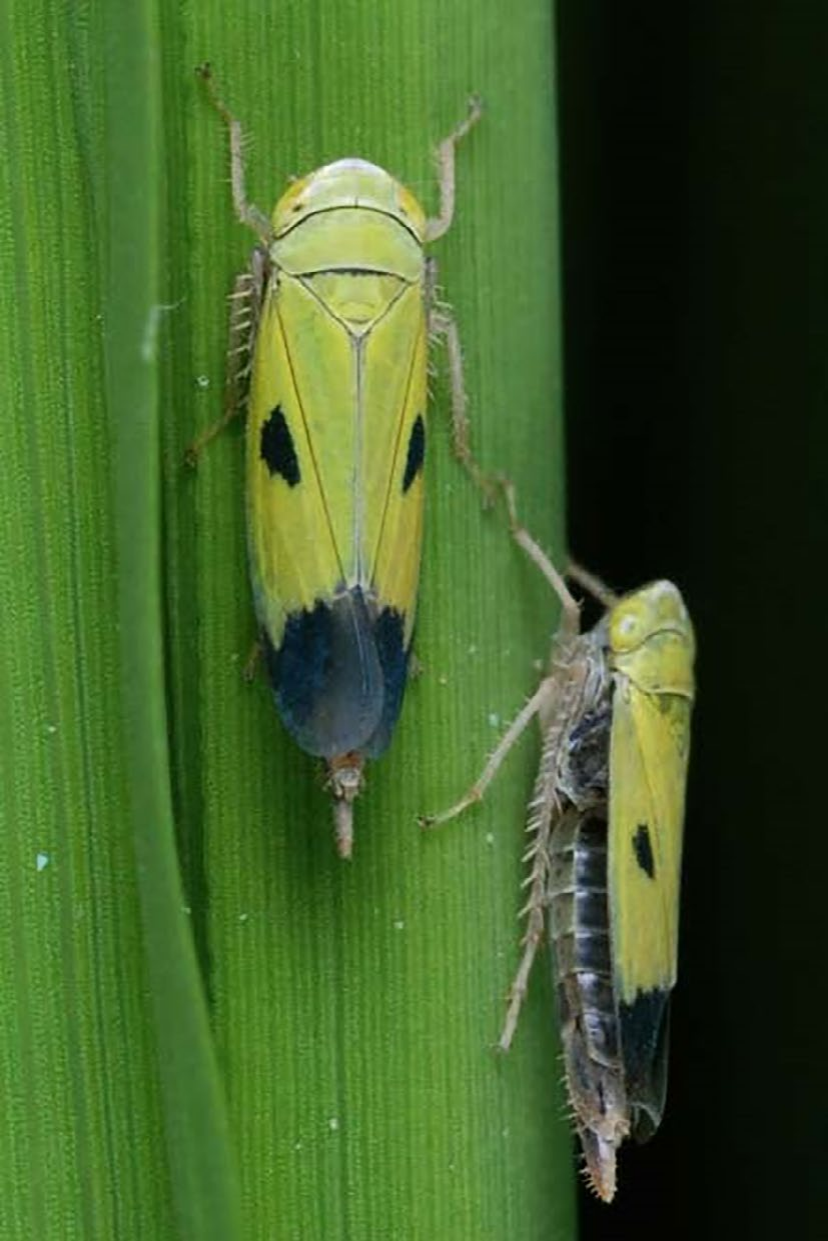
The green leafhopper, *Nephotettix virescens* (photo courtesy of IRRI Knowledge Bank)

The present study examines the microbiomes of green leafhopper, *Nephotettix virescens* (Figure 1), colonies selected on near‐isogenic rice lines (NILs) with one or two genes for resistance to *Nephotettix cincticeps* (see below), and on the susceptible recurrent parent, T65. *Nephotettix virescens* is a polyphagous leafhopper (Khan, Hibino, Aguiero, Daquioag, & Opina, 1991) that is a considerable pest of rice (*Oryza sativa*) in South and South‐east Asia. The leafhopper occasionally causes mechanical damage to rice plants, but more importantly transmits tungro rice viruses (Azzam & Chancellor, 2002; Khan et al., 1991). Beginning in the 1970s, a number of studies indicated that leafhopper populations are variously affected by resistance factors present in traditional rice varieties (Fujita et al., 2013). Although the mechanisms remain largely undetermined, these resistance factors reduce leafhopper feeding, nymph development, and egg laying (Asano et al., 2015; Horgan et al., 2019). Currently, more than 24 loci with genes for resistance to leafhoppers (*Nephotettix* spp. and *Recelia dorsalis*) have been identified (Fujita et al., 2013) and a number of these genes have been introgressed into the *japonica* rice variety T65 using marker‐assisted selection (Horgan et al., 2019). In our experiments, we used monogenic near‐isogenic rice lines (NILs) carrying either the *GRH2* or *GRH4* gene loci (henceforth *GRH2*‐NIL and *GRH4*‐NIL, respectively) with resistance to *N. cincticeps*, and a line carrying both genes together (henceforth *GRH2/GRH4*‐PYL [we use “PYL” as an abbreviation for the breeding term “pyramided,” which indicates a breeding line or NIL with ≥2 resistance genes]). Both *GRH2* and *GRH4* were first identified from DV85 using *N. cincticeps* in phenotyping studies. Resistance mechanisms associated with *GRH2* and *GRH4* have not been fully elucidated; however, Asano et al. (2015) found that genes for several types of proteinase inhibitor and several genes of the cytochrome P450 family were expressed in a *GRH2/GRH4*‐PYL infested by *N. cincticeps*. Furthermore, genes associated with the production of volatiles, particularly sesquiterpenes, were upregulated during *N. cincticeps* attack (Asano et al., 2015). Previous studies have indicated that the pyramided line, *GRH2/GRH4*‐PYL, is highly resistant to *N. virescens* (Horgan et al., 2018, 2019).

By continually monitoring the fitness of *N. virescens* on *GRH2/GRH4*‐PYL over successive generations, Horgan et al. (2018) noted that whereas partial virulence was achieved within ten generations (i.e., leafhopper feeding efficiency, survival, and weight gain equal to that on susceptible varieties), leafhoppers required several further generations of selection to achieve complete virulence (i.e., egg laying equal to that on susceptible varieties). We therefore used colonies only after 20 generations of selection to insure complete virulence adaptation in our study. We assessed the microbiomes of these adapted colonies to identify possible microbiome associations with virulence of the insect host. Horgan et al. (2018) have also shown that *N. virescens* colonies successively reared on the monogenic resistant lines (*GRH2*‐NIL and *GRH4*‐NIL) were capable of surviving and developing on the pyramided line *GRH2/GRH4*‐PYL without any prior exposure to that line (henceforth phase I selection). Therefore, we expected that aspects of microbiome community structure would be similar among colonies reared on *GRH2*‐NIL, *GRH4*‐NIL, and *GRH2/GRH4*‐PYL, but different from colonies reared on T65. Without prior evidence to suggest which bacterial taxa might influence virulence or how the microbiomes might react to host plant resistance, we focused on two response levels. Firstly, we examined the overall composition of the microbiomes from the different colonies. Secondly, we looked at the abundance and proportional representation of different taxa in the leafhopper samples to detect significant host plant effects. To further test the hypothesis, we examined changes in key bacterial taxa (identified after phase I selection) among leafhoppers when moved to novel resistant hosts for further selection (phase II selection). In particular, we predicted that switching leafhoppers from a susceptible to a more resistant host would produce changes in the abundance of the key OTUs identified from phase I. To relate potential changes in the microbiome to further adaptation, we continuously monitored the leafhopper colonies for survival, weight gain, and egg laying throughout phase II selection. We also assessed their ability to damage the host plant as populations became increasingly virulent. We discuss our results in light of current knowledge of the microbiomes of insect herbivores and of the mechanisms of virulence adaptation in phloem‐feeding leafhoppers.

## 2 MATERIALS AND METHODS

### 2.1 Plant materials

We obtained DV85 and T65 from the Germplasm Bank at the International Rice Research Institute (IRRI) in the Philippines. The resistant lines that we used, *GRH2*‐NIL, *GRH4*‐NIL, and *GRH2/GRH4*‐PYL, were BC_6_F_5_ generations selected using Simple Sequence Repeat markers associated with the target loci during repeated backcrossing of the donor variety DV85 and the recurrent parent T65 (Horgan et al., 2018, 2019). Seeds of the NILs were bulked‐up in a screen‐house at IRRI during the dry season when temperatures were coolest.

### 2.2 Leafhopper colonies and Phase I selection

In this study, we used greenhouse colonies derived from five initial *N. virescens* populations. These were from original collections made in rice fields at Laguna, Batangas, Quezon, Rizal, and San Pablo (Luzon Island, Philippines). The colonies were each selected on the four rice lines (henceforth “natal” hosts) for 20 generations (5 populations × 4 natal hosts = 20 colonies). Details of selection and the nature of the selected colonies (their ability to develop on natal hosts) are described in detail by Horgan et al. (2018). Briefly, selection was conducted as follows.

At two to three generations after field collection, the five populations reared on the rice variety TN1 were each split into four parts (with >200 adults each) and placed in insect cages (120 × 60 × 60 cm, height × length × width [*H* × *L* × *W*]) on the natal hosts, T65, *GRH2*‐NIL, *GRH4*‐NIL, and *GRH2/GRH4*‐PYL. Feeding plants (the natal hosts) of 30 days after sowing (DAS) in size #6 plastic pots (15 × 15 cm, height × rim diameter [*H* × *D*]) filled with saturated paddy soil were supplied ad libitum. The plants were changed every 2 weeks. The colonies were continuously reared on the hosts for 20 generations and were monitored for virulence adaptation during selection (see Horgan et al., 2018). For the purposes of the present study, we present the results of virulence monitoring from populations prior to selection and after 20 generations of Phase I selection.

### 2.3 Changes in virulence during phase I selection

Prior to selection and after 20 generations of selection, leafhoppers from each of the colonies (*N* = 5) were assessed for comparative fitness on their natal hosts T65, *GRH2*‐NIL, *GRH4*‐NIL, or *GRH2/GRH4*‐PYL. After 20 generations of selection, the leafhoppers were also assessed for their abilities to survive, develop, and lay eggs on the pyramided line *GRH2/GRH4*‐PYL. Fitness was assessed through nymph survival, adult survival, and egg‐laying bioassays.

To assess adult and nymph survival, newly emerged gravid females (isolated as fifth instars to ensure they were unmated) and nymphs, respectively, were collected from each colony (5 adults or 10 neonates per plant) and placed on undamaged natal host plants (20 DAS). The test plants were grown in size #0 pots (5 × 5 cm, *H* × *D*) under acetate insect cages (45 × 5 cm, *H* × *D*). After 15 days, the number of survivors on each plant and their development stages were recorded. The survivors were collected and dried in a forced draft oven at 60°C for 3 days before being weighed. The feeding plants were also dried and weighed.

Egg laying was assessed by introducing mated, gravid females (2 females) to 20 DAS plants grown in size #0 pots under insect cages (dimensions as above). The females were allowed to oviposit for 5 days after which the plants were collected and dissected to count the eggs.

All bioassays (for phase I and phase II [see below]) were conducted on a greenhouse bench (temperature: 25–37°C; 12 hr:12 hr, day:night) using a randomized block (=origin) design. Colonies of different origin were not assessed as a treatment during the selection studies but were used to gain increased external validity from the selection experiments (Ferrater et al., 2013).

### 2.4 Leafhopper selection on *GRH2/GRH4*‐PYL (phase II selection)

Adult leafhoppers (150–200 pairs) were collected from colonies that had been selected on *GRH2*‐NIL and *GRH4*‐NIL for 20 generations during phase I selection or were without exposure to any resistance loci (maintained for 20 generations on T65). The adults were transferred to cages (dimensions as above) with *GRH2/GRH4*‐PYL as feeding plants (i.e., a novel natal host), allowed to oviposit, and nymphs allowed to develop. These new colonies (five for each original natal host, *GRH2*‐NIL, *GRH4*‐NIL, and T65 = 15) were continuously exposed to *GRH2/GRH4*‐PYL for six generations. A series of control colonies that were continuously reared on their original natal hosts was also maintained (5 colonies × 4 original natal hosts = 20). Therefore, 35 colonies were simultaneously maintained during the experiment.

At each generation, the colonies were monitored to assess nymph survival, adult survival, and egg laying on *GRH2/GRH4*‐PYL using the bioassays described above.

### 2.5 Leafhopper sampling for microbiomes

**Figure 2:**
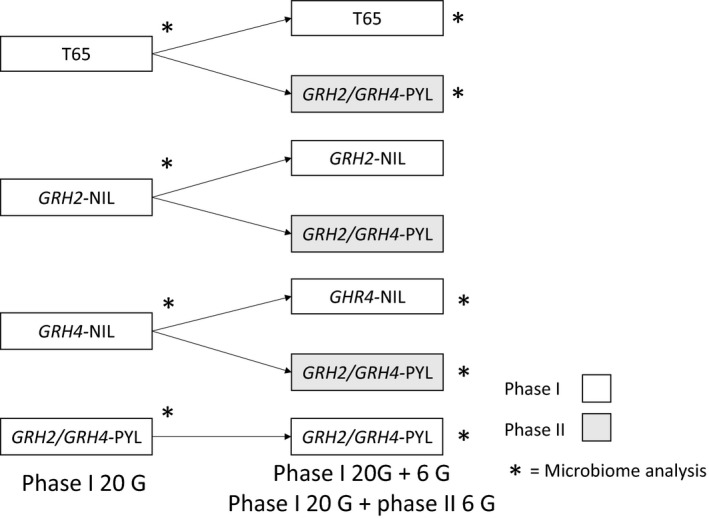
Details of selection experiments with green leafhopper colonies and microbiome sampling. Colonies were selected for 20 generations (G) on each of four rice lines and sampled for microbiome at 20 G. Selection on the natal hosts was continued for a further 6 G with microbiome sampling of T65‐, *GRH4*‐NIL‐, and *GRH2/GRH4*‐PYL‐selected colonies at 26 G. Colonies that were switched to *GRH2/GRH4*‐PYL after 20 G (phase II selection) were sampled for microbiome after 6 generations (simultaneously with 26 G phase I)

We examined the microbiomes of each colony after 20 generations [G] of phase I selection on their natal hosts (5 locations × 4 natal hosts = 20 colonies, henceforth phase I at 20 generations [phase I 20 G]). We also examined the microbiomes of 15 of these colonies after six further generations (phase I 20 G + 6 G) on their original natal hosts (T65, *GRH4*‐NIL, and *GRH2/GRH4*‐PYL). We did not examine the microbiome of the five colonies on *GRH2*‐NIL after 26 generations of selection because the colonies demonstrated virulence against *GRH2/GRH4*‐PYL that was intermediate between the virulence of *GRH4*‐NIL‐selected and *GRH2/GRH4*‐PYL‐selected colonies, thereby adding little value to the analysis. We further examined the microbiomes of 10 colonies that had been transferred from T65 or *GRH4*‐NIL to *GRH2/GRH4*‐PYL for six generations (phase I 20 G + phase II 6 G; Figure 2).

Three adult female leafhoppers (≤10 days after emergence) were randomly selected from each colony (phase I 20 G: 4 natal hosts × 5 colonies = 20 samples, phase I 20 G + 6 G: 3 natal hosts × 5 colonies = 15 samples; phase I 20 G + phase II 6 G: 2 natal hosts × 5 colonies = 10 samples; total = 45 samples: Figure 2). The sampled leafhoppers were surface sterilized using 70% ethanol for 1 min and placed in clean 1.5 ml microtubes on ice.

### 2.6 Extraction of sample DNA

Samples were homogenized in 2× CTAB extraction buffer and 2 µl of β‐mercaptoethanol using a micropestle. The homogenized mixture was treated with 2.5 µl of ProteinaseK (20 mg/ml) and kept for lysis at 55°C for 1 hr. The samples were intermittently mixed every 15 min. After the lysis step, the samples were cooled briefly and an equal volume of chloroform:isoamyl alcohol (24:1) (500 µl) was added and centrifuged at 5,000 × *g* for 15 min at 4°C. After centrifugation, the top aqueous phase was carefully transferred to another 1.5‐ml microtube. The top aqueous phase containing the DNA was precipitated by adding 500 µl of isopropanol and 50 µl of 3 M sodium acetate, mixed gently, and incubated at −20°C overnight. After overnight incubation at −20°C, the DNA was precipitated by centrifuging at 5,000 × *g* for 15 min at 4°C. The supernatant was decanted and the pellet was washed twice with 70% ethanol and air‐dried. The pellet was then dissolved in TE buffer of 50 µl + 1 µl of RNAse (20 mg/ml) and incubated at 37°C for 30 min.

After incubation, the DNA was precipitated using 10 µl of 3 M sodium acetate and 200 µl of absolute ethanol and incubated overnight at −20°C. The DNA was then precipitated by centrifuging at 5,000 × *g* for 15 min at 4°C. The supernatant was decanted, and the pellet was washed with 70% ethanol and air‐dried. The DNA pellet was then dissolved in 50 µl of TE buffer and quantified using 0.8% agarose gel and a NanoDrop 2000 UV‐Vis Spectrophotometer.

### 2.7 Confirmation and sequencing of bacterial symbionts using bacterial specific primers

The extracted sample DNA was confirmed for the presence of bacterial symbionts by PCR using bacterial specific primers (i.e., Univ‐0008‐a‐S‐19f and Univ‐1528‐a‐A‐17r). Details of primer pairs and their sequences are provided in Table 1. The PCR amplification was performed in a G‐Storm GS1 Thermal Cycler. The 20‐μl of PCR mixture contained 1× PCR buffer, 1.5 mM MgCl_2_, BSA 10 μg/ml, 0.2 mM dNTP, 5 μM forward and reverse primer each and 1 U Taq polymerase (Takara, Japan), and 100 ng of genomic DNA as a template. The PCR products were resolved in 1% agarose gels by means of electrophoresis at 150 V for 1 hr in 0.5 × Tris–borate–EDTA buffer. The gels were stained with GelRed™ (Biotium) and photographed under ultraviolet light in a Bio‐Rad Gel Doc™ XR + System. The amplified DNA fragment (50 ng/μl) was submitted to Eureka genomics service, USA, for Illumina‐based sequencing of the V5 region.

**Table 1:** Primers used for confirmation of bacterial symbionts

Primer	Sequence	Product size	Blast hit
Univ‐0008‐a‐S‐19f	GAGTTTGATCCTGGCTCAG	1,538	rRNA‐16s ribosomal RNA
Univ‐1528‐a‐A‐17r	AAGGAGGTGATCCAGCC	1,538	rRNA‐16s ribosomal RNA

### 2.8 Sequence processing and clustering

Sequenced reads were quality filtered, dereplicated, abundance sorted, clustered, and chimera removed in the UPARSE pipeline (Edgar, 2013). Quality filtering of reads was conducted with a maximum expected number of errors of less than 0.5, which assumes that the average error of each base in each read is 0.5, and additionally, 8‐bp barcodes at the 5′ end were removed, thus producing 143 high‐quality base reads. Singletons were also removed. For filtering of chimeras, UCHIME (Edgar, Haas, Clemente, Quince, & Knight, 2011) was used against the RDP classifier training database (v9). Raw sequencing reads were mapped to the chimera filtered database at the 97% similarity threshold and were used to define the OTUs. A total of 264 OTUs along with the mock were obtained. OTUs were trimmed off to remove mock as well as OTUs with a total count of <10, and a final list of 227 OTUs were obtained. For the classification of the bacterial OTUs, megablast (NCBI Resource Coordinators, 2016) was used against the NCBI database. Taxonomy assignment was carried out through manual curation of the blast output with criteria based on the highest bit score, greater than 98% query coverage and at least 97% identity score. OTUs, which failed to suffice all of the criteria, were considered unclassified.

### 2.9 Data analyses

Because greenhouse conditions, particularly temperature, varied throughout the course of the selection and associated monitoring experiments, leafhopper development, and egg laying fluctuated between generations. We therefore present fitness results relative to leafhoppers feeding on the susceptible recurrent parent (T65) for phase I and relative to *GRH2/GRH4*‐PYL‐selected leafhoppers for phase II; however, full results are also included in the Tables S1–S8. Relative fitness of leafhoppers during selection was examined using univariate general linear models (GLM). Similarly, virulence on the respective natal hosts and on *GRH2/GRH4*‐PYL was examined using GLMs after 20 generations of phase I selection. The factor “origin” (referring to the original collection sites in the Philippines: levels = Batangas, Laguna, Quezon, Rizal, and San Pablo) was initially included in all models, but was removed where it had no significant effect. The covariate “rice plant weight” was also initially included in the analyses but removed where it had no effect.

The virulence of leafhoppers on *GRH2/GRH4*‐PYL during each generation of phase II selection was examined using repeated measures GLM with generation as the repeated measure and original natal host (phase I selection) as the main factor. The factor “origin” was initially included in all models, but was removed where there was no significant effect.

The microbiomes of leafhoppers after Phase I and Phase II selection were initially examined at the community level. Prior to statistical analysis, data were square‐root transformed (Council et al., 2016). Permutational analysis of variance (PERMANOVA: Anderson, 2001) was used to analyze differences in microbiota composition, at both the OTU and the genus levels. PERMANOVA is a robust semiparametric method that uses permutation techniques to calculate *p*‐values. For Phase I‐selected leafhoppers, the factors included in the PERMANOVA analysis were “origin” (random) and “natal” (fixed).

For phase I 20 G + 6 G and phase I 20 G + phase II 6 G leafhoppers, two factors, “origin” (random) and “transition” (fixed), were included in the analysis. “Transition” was created to test the effect of maintaining phase I selected colonies on the same host, or switching a colony from one host plant to another during six generations. The factor “transition” had five levels: 0–0, T65 to T65 (i.e., 0 locus to 0 locus); 0–2, T65 to *GRH2/GRH4*‐PYL (i.e., 0 locus to 2 loci); 1–1, *GRH4*‐NIL to *GRH4*‐NIL (i.e., 1 locus to 1 locus); 1–2, *GRH4*‐NIL to *GRH2/GRH4*‐PYL (i.e., 1 locus to 2 loci); and 2–2, *GRH2/GRH4*‐PYL to *GRH2/GRH4*‐PYL (i.e., 2 loci to 2 loci).

PERMANOVA pairwise tests were conducted to analyze differences between levels of statistically significant factors. The PERMDISP routine was used to examine homogeneity of dispersions (based on mean distance to group centroids), to ensure that dispersions were constant among groups (Anderson, 2006). Nonmetric multidimensional scaling (MDS) was used to visualize between‐group differences in microbiota composition. Similarity, percentage analysis (SIMPER) was used to identify which OTUs or taxa contributed most to the dissimilarities between groups.

To restrict our focus to bacteria with a potential functional role in virulence, we examined the proportion of prominent OTUs (present among all source colonies and on all host plants, and represented in >40% of the samples) in samples after phase I selection using multivariate GLM. Similarly, the proportions of distinct OTUs (see below) assigned to the *Candidatus sulcia* clade were examined using multivariate GLM. Abundances of each taxon were examined by univariate GLM. The abundance of six taxa (see below) after phase II selection was analyzed by univariate GLM with initial natal host and transition to new host as main factors. Origin was included as a blocking factor.

PERMANOVA, PERMDISP, MDS, and SIMPER analyses were carried out using PRIMER software (v.6.1.16) with the PERMANOVA + add‐on (v.1.0.6). Multivariate and univariate GLMs were carried out using SPSS v.22 (SPSS). Data residuals were plotted to test for normality and homogeneity of residuals following the application of all GLMs. Where residuals were not normal or homogeneous, we transformed the data as indicated with the results.

## 3 RESULTS

### 3.1 Virulence after phase I selection

**Figure 3:**
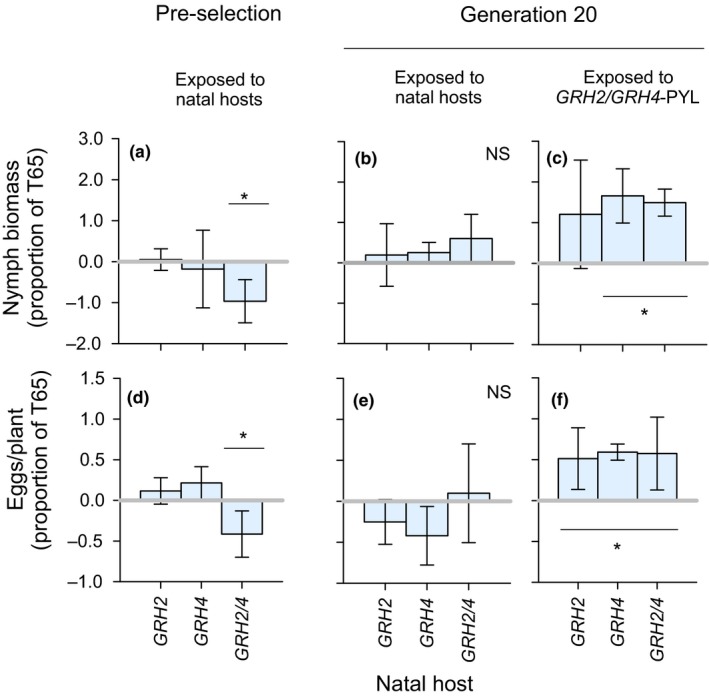
Results from fitness bioassays conducted on colonies (a, d) prior to selection and (b, c, e, f) after 20 generations of selection. Nymph biomass (dry weight) (a–c) and the number of eggs laid (d–f) were monitored for preselection leafhoppers (reared on TN1) and exposed to their natal hosts (a, d) and for leafhoppers after 20 generations of selection exposed to their natal hosts (b, e) or exposed to the pyramided resistant line *GRH2/GRH4*‐PYL. Asterisks indicate significant differences (Tukey tests: *p* ≤ .05; *N* = 5 colonies) from T65 = natal host (indicated by gray line at *y* = 0), NS = no significant host effect. Standard errors are indicated. See Table S1 for full results from virulence monitoring

Prior to selection, leafhoppers that were recently collected from the field sites were capable of developing on *GRH2*‐NIL and *GRH4*‐NIL, but had poor survival, development, and egg laying on *GRH2/GRH4*‐PYL (Figure 3a,d; Table S1). After 20 generations of selection, all colonies had fully adapted to feed, survive, develop, and lay eggs on their respective natal hosts (Figure 3b,e; Table S1). Furthermore, leafhoppers selected on both the monogenic (*GRH2*‐NIL and *GRH4*‐NIL) and pyramided (*GRH2/GRH4*‐PYL) lines had a greater capacity to develop and lay eggs on the pyramided line after 20 generations compared to those reared continuously on T65 (Figure 3c,f; Table S1).

### 3.2 Phase II selection on *GRH2/GRH4*‐PYL

**Figure 4:**
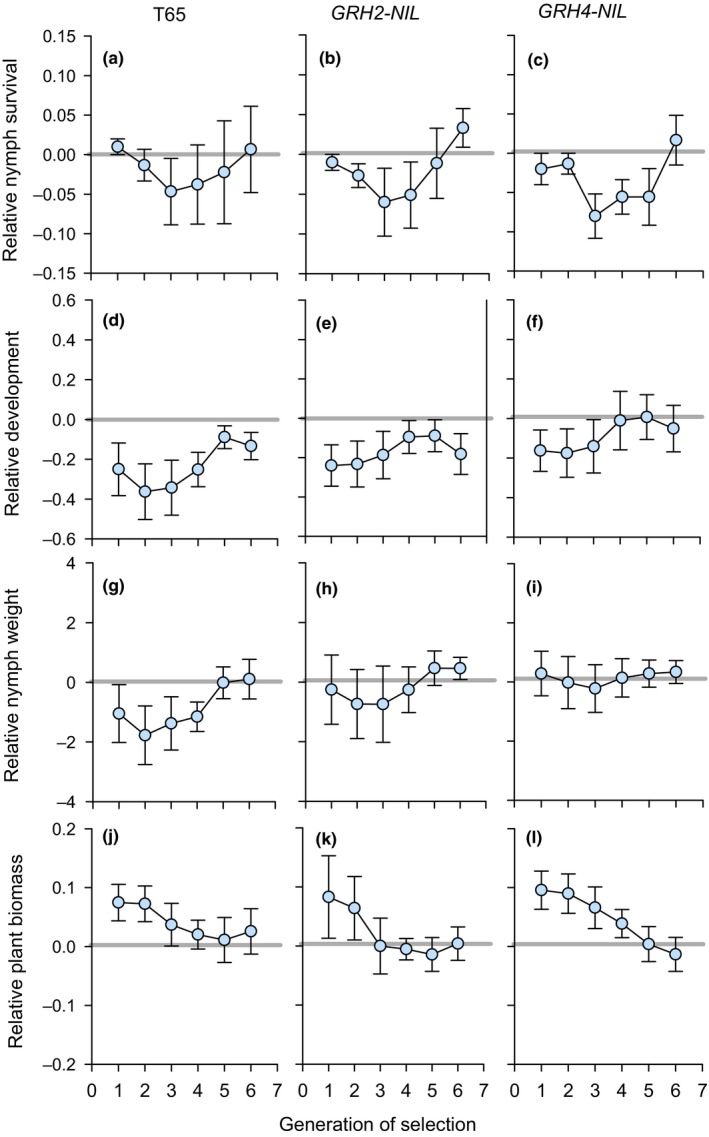
(a–c) Leafhopper nymph survival, (d–f) nymph development to adult, (g–i) nymph biomass, and (j–l) biomass of *GRH2/GRH4*‐PYL plants after nymph feeding during selection on *GRH2/GRH4*‐PYL for six generations. Selection was initiated using colonies that were previously selected (phase I) on (a, d, g, j) T65, (b, e, h, k) *GRH2*‐NIL, and (c, f, i, l) *GRH4*‐NIL for 20 generations. Leafhopper responses are indicated relative to responses by *GRH2/GRH4*‐PYL‐selected colonies (phase I) without switching the natal host during six contemporaneous generations (indicated by gray line at *y* = 0). Standard errors are indicated (*N* = 5 colonies). See also Table S2

Leafhoppers that had been previously selected on T65, *GRH2*‐NIL, and *GRH4*‐NIL (phase I) followed largely similar patterns in virulence over six generations of phase II selection on *GRH2/GRH4*‐PYL (Figure 4). During phase II selection, nymph survival was similar among leafhoppers from all colonies (Figure 4a–c; Table S2). Nymph development (Figure 4d–f) and nymph weight gain (Figure 4g–i) gradually improved over the six generations (development: *F*‐generation = 39.580, *p* ≤ .001; biomass: *F*‐generation = 49.909, *p* ≤ .001) resulting in progressively more severe damage to rice plants from the pyramided line (*F*‐generation = 124.966, *p* ≤ .001) in bioassays conducted at each successive generation (Table S2; Figure 4j–l). During phase II selection, adult survival, adult biomass, and egg laying on *GRH2/GHR4*‐PYL were similar irrespective of the phase I natal plant, including *GRH2/GRH4*‐PYL (Table S2). Only nymph development showed a significant generation × host interaction (*F*_15,80_ = 2.327, *p* ≤ .01) because of greater final (generations 4, 5, and 6) adaptation to survive on *GRH2/GRH4*‐PYL by colonies that had been selected on *GRH4*‐NIL during phase I (Figure 4d–f).

### 3.3 OTU assembly

Sequencing of 16s rRNA‐ V5 amplicons from the phase I 20 G, Phase I 20G + 6 G, and Phase I 20 G + phase II 6 G samples yielded 60,232–281,854 counts per insect sample. Counts were uniform among the samples. A total of 227 and 208 OTUs were assigned to phase I and phase II samples, respectively (total = 227). Full details of the OTUs and their corresponding taxa are presented in Table S3.

### 3.4 Leafhopper microbiome after phase I selection

**Figure 5:**
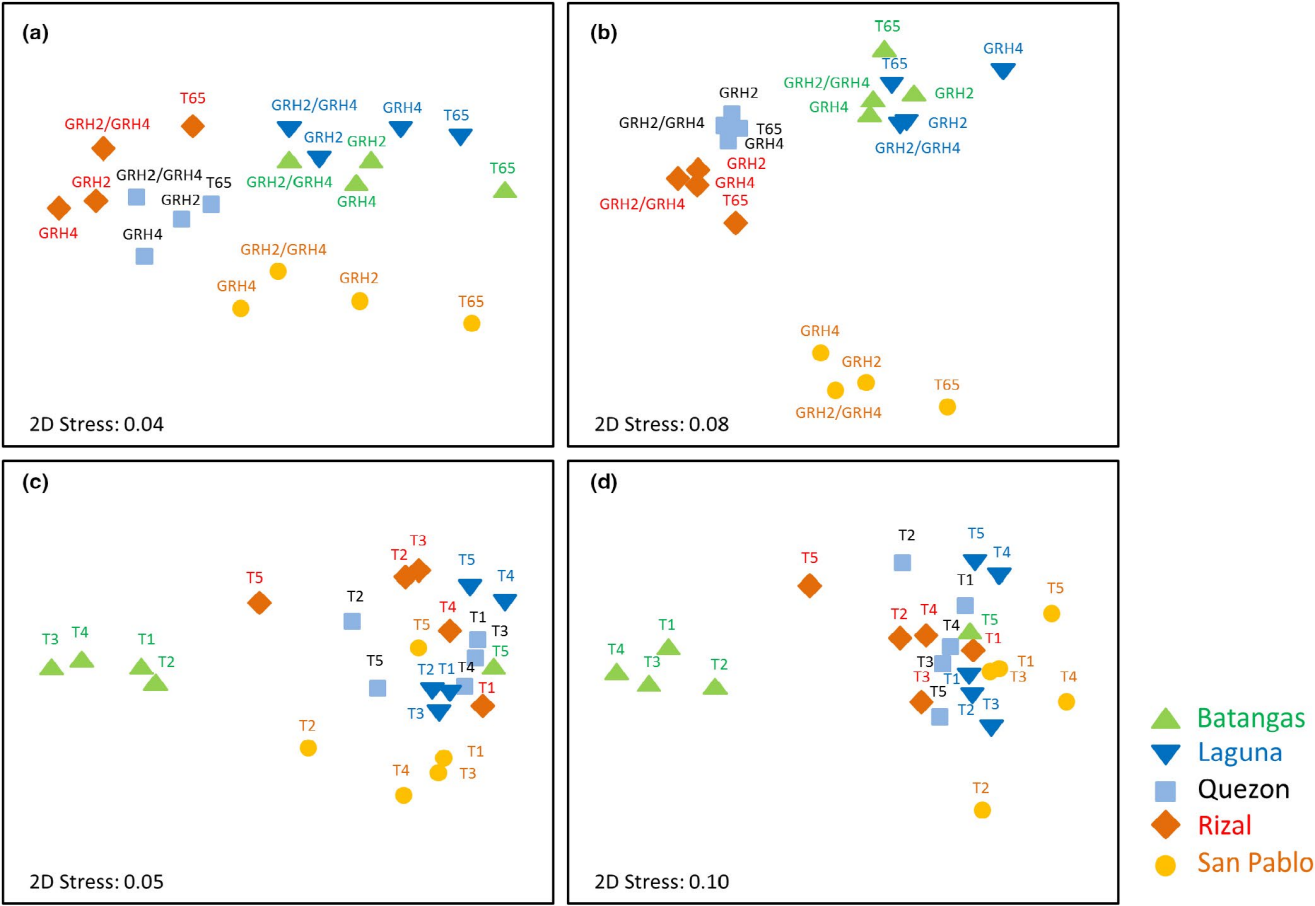
Nonmetric multidimensional scaling (MDS) showing ordination of the microbiome communities associated with leafhoppers after (a, b) phase I selection (phase I 20 G) with (c, d) relative changes in microbiomes after six further generations on the same natal host (phase I 20 G + 6 G, 0–0, 1–1, and 2–2) or after phase II selection (phase I 20 G + phase II 6 G, 0–2, and 1–2). Plots represent abundance data at (a) the OTU level, and (b) the genus level after phase I selection. Symbols indicate locations and labels indicate (a, b) natal hosts or (c, d) “transitions,” 0–0, 0–2, etc., between host plant species, where 0 = T65, 1 = *GRH4*‐NIL, and 2 = *GRH2/GRH4*‐PYL. For further details, see Table S7

At the OTU level, both “location” and “natal” host had a significant impact on the microbiome community of leafhoppers (Pseudo‐*F* = 18.966, *p* = .001 and Pseudo‐*F* = 6.0432, *p* = .002, respectively). “Origin” had a greater effect than “natal” at the community level as visualized in the MDS ordination plot (Figure 5a). PERMANOVA pairwise test results indicated that microbiomes were significantly different between all locations (all showing *p*‐values ≤ .05), except between Batangas and Laguna (*t* = 0.929, *p* = .557; Figure 5a). Pairwise tests between levels of the “natal” factor showed significant differences between T65 and all other rice lines (all with *p*‐values ≤ .05). More than 80% of the pairwise cumulative dissimilarity between levels was significantly different for both “origin” and “natal” factors because of differences in the abundance of six OTUs (i.e., numbers 1, 8, 10, 12, 17, and 54) each identified as belonging to the *Canditatus sulcia* clade (Tables S4 and S5).

**Figure 6:**
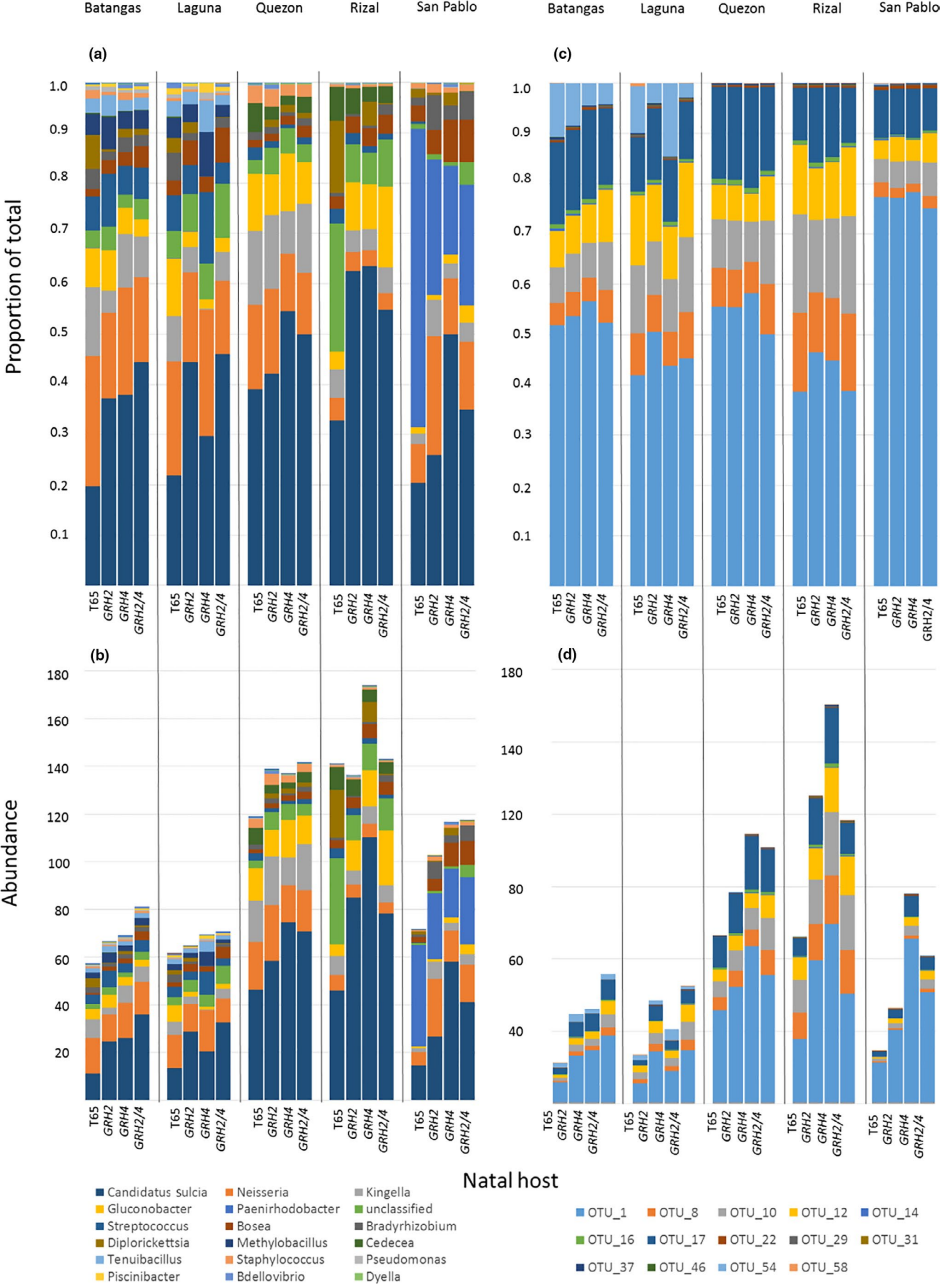
(a) The proportion of total OTUs represented by 16 prominent taxa (i.e., most abundant in phase I 20 G samples and occurring in samples from all five origins) with (b) the abundance of each OTU in the samples. The composition of *Candidatus sulcia*, which consisted of 14 OTUs, is indicated by (c) the proportion of each OTU in the samples and (d) the abundance of the OTUs in the samples

At the genus level, the microbiome community was significantly affected by “origin” (Pseudo‐*F* = 14.607, *p* = .001) but not by “natal” host (Pseudo‐*F* = 1.338, *p* = .239; Figure 5b). Similarly to the OTU level, pairwise differences between locations were all significant (all showing *p*‐values ≤ .05), except between Batangas and Laguna (*t* = 1.506, *p* = .198). ANOSIM showed that the average dissimilarities between San Pablo and every other location were higher than other comparisons (Table S6). This was due to *Paenirhodobacter* spp., which contributed ≥22.86% to the dissimilarity with other locations (Figure 6; Table S6). Significant PERMANOVA results in phase II were not affected by data dispersion, as confirmed by PERMDISP tests, which all showed nonsignificant *p*‐values (>.05).

### 3.5 Changes in microbiota during phase I selection

Only 177 OTUs (39 suggested taxa and unclassified groups) were associated with leafhoppers from each source colony and from each host plant at phase I 20 G (Table 2; Figure 6). Origin, but not natal host affected the relative proportions of OTUs across samples (multiple GLM: natal host: *F*_9,36_ = 1.196, *p* = .412; origin: *F*_16,36_ = 4.218, *p* = .001). Origin significantly affected the proportion of 26 taxa (between subject effects), whereas natal host affected only six taxa (*Bosea*, *Candidatus sulcia*, *Dyadobacter*, *Dyella*, *Mycobacterium*, and *Sandaracinus*; Figure 6a; Table 2).

**Table 2:** Taxa with corresponding number of OTUs from each of the five colonies and on all four rice lines with >40% occurrence across samples

Phylum	Genus	Number of OTUs	Abundance at generation 20	Abundance *F*‐natal†	Abundance *F*‐origin†	Proportion of samples	Proportion *F*‐natal^a^^,^ †	Proportion *F*‐origin^a^^,^ †
Actinobacteria	*Curtobacterium*	1	5,867	1.420ns	202.222***	0.51	0.569ns	14.382***
	*Mycobacterium*	1	367	5.911*	10.039***	0.98	3.997*	5.375**
Bacteroidetes	*Fluviicola*	1	40	2.779ns	2.017ns	0.73	1.936ns	2.625ns
	*Dyadobacter*	1	568	2.177ns	4.372*	0.98	5.220**	8.606**
	*Candidatus sulcia*	14	903,711	13.413***	24.257***	1.00	7.161***	8.733**
Firmicutes	*Aeribacillus*	1	9	0.567ns	0.559ns	0.42	0.508ns	0.516ns
	*Anoxybacillus*	2	8,038	0.614ns	105.759***	0.60	0.337ns	20.879***
	*Tenuibacillus*	1	17,221	0.221ns	78.297***	0.96	0.564ns	15.332***
	*Ammoniphilus*	1	104	0.146ns	0.207ns	0.51	1.326ns	0.640ns
	*Staphylococcus*	7	27,864	0.117ns	31.283***	1.00	1.197ns	28.455***
	*Streptococcus*	7	60,130	1.474ns	25.711***	0.98	0.529ns	10.503***
Proteobacteria	*Piscinibacter*	2	4,379	0.483ns	57.715***	0.64	0.396ns	15.186***
	*Gluconobacter*	11	145,132	0.208ns	9.204***	1.00	0.119ns	3.883*
	*Bartonella*	1	328	1.999ns	1.670ns	0.69	0.910ns	4.254*
	*Bdellovibrio*	1	4,754	0.460ns	99.158***	0.49	0.354ns	18.294***
	*Bosea*	1	75,557	11.342***	12.707***	1.00	4.737*	5.268**
	*Bradyrhizobium*	2	43,345	1.403ns	1.786ns	1.00	0.737ns	1.796ns
	*Burkholderia*	1	98	0.688ns	6.244*	0.71	0.230ns	1.064ns
	*Limnohabitans*	1	122	1.020ns	0.922ns	0.51	1.033ns	0.942ns
	*Diplorickettsia*	9	49,100	0.548ns	0.107ns	0.98	2.225ns	1.236ns
	*Cedecea*	7	44,304	1.598ns	24.233***	0.91	2.838ns	20.158***
	*Klebsiella*	2	30	0.825ns	0.390ns	0.60	1.347ns	0.673ns
	*Tepidiphilus*	1	1,997	0.981ns	4.016*	1.00	0.922ns	4.402*
	*Hyphomicrobium*	1	26	0.110ns	0.266ns	0.69	0.001ns	3.280*
	*Methylobacillus*	2	24,494	1.024ns	76.176***	0.51	0.593ns	14.139***
	*Methylotenera*	1	2,081	0.243ns	8.023**	1.00	0.692ns	9.568***
	*Acinetobacter*	1	42	0.564ns	0.278ns	0.67	0.567ns	0.491ns
	*Kingella*	7	151,325	0.865ns	3.986*	1.00	1.501ns	8.681**
	*Neisseria*	14	264,243	0.355ns	7.331**	1.00	0.891ns	12.898***
	*Haemophilus*	5	115	0.640ns	0.713ns	0.93	1.001ns	0.843ns
	*Pseudomonas*	6	4,798	1.672ns	20.247***	1.00	0.635ns	14.630***
	*Paenirhodobacter*	8	118,940	3.130ns	94.325***	0.76	0.999ns	11.635***
	*Georgfuchsia*	1	101	0.607ns	13.288***	0.47	1.428ns	2.946ns
	*Sandaracinus*	1	171	4.825*	10.616***	0.96	3.437*	10.927***
	*Nevskia*	2	1,082	1.047ns	0.572ns	0.60	1.028ns	0.720ns
	*Sphingomonas*	1	85	1.214ns	1.819ns	0.87	1.054ns	2.063ns
	*Dyella*	1	2,478	13.456***	39.287***	1.00	7.140***	27.527***
	*Xanthomonas*	1	690	0.097ns	66.965***	0.42	0.666ns	24.970***
	*Rubrivivax*	1	46	0.321ns	0.221ns	0.58	0.602ns	0.559ns
Unclassified		47	223,616	0.458ns	23.092***	1.00	1.039ns	18.217***

Note

Counts representing each taxon together with the proportion of total reads represented by each taxon at generation 20 are indicated with corresponding *F*‐values (natal host and origin) for each taxa (univariate [abundance] and multivariate [proportions] GLMs; see also Figure 5a,b). Full details of all OTUs from both selection phases are indicated in Table S3.

a Based on multivariate GLM.

^†^ns = *p* ≥ .05, *p ≤ .05, **p ≤ .01, ***p ≤ .001; DF natal host = 3,12, DF origin = 4,12.

**Figure 7:**
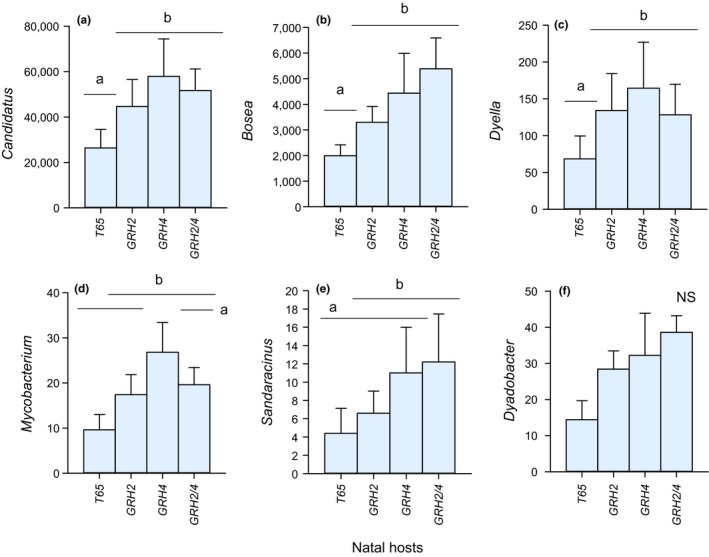
The relative abundance of key bacterial genera associated with the microbiomes of leafhoppers reared for 20 generations on four natal hosts. The genera are from the (a) *Candidatus sulcus* clade, (b) *Bosea*, (c) *Dyella*, (d) *Mycobacterium*, and (e) *Sandaracinus*. Data for (f) *Dyanobacter* are also indicated: The abundance of this genus was not associated with host plant, but the proportional representation by the taxon in the samples was significantly higher in leafhoppers on *GRH2/GRH4*‐PYL than on T65 (see Table 2). Error bars are indicated, lowercase letters indicate homogenous groups (Tukey, *p* ≤ .05, *N* = 5 colonies)

Abundances varied significantly between origins for 26 of the 39 taxa (Table 2; Figure 6b). However, the abundance of only five taxa varied significantly based on natal host (Table 2, Figure 6b). Each of these five taxa (*Bosea*, *Candidatus sulcia*, *Dyella*, *Mycobacterium*, and *Sandaracinus*) were consistently more prevalent among leafhoppers selected on *GRH4*‐NIL or *GRH2/GRH4*‐PYL compared to those reared on the susceptible recurrent parent T65 (Figure 7), thereby largely matching patterns in leafhopper virulence (see Figure 3).

The relative proportions of individual *Candidatus sulcia* OTUs were affected by origin; only OTUs 14, 46, and 58 had similar proportions across origins (Figure 6c; Table 3). The proportions of OTUs 14, 16, 29, 46, 54, and 58 making up the clade were not affected by natal host; among the remaining OTUs, proportions were most similar for leafhoppers on T65 and *GRH2/GRH4*‐PYL and did not match patterns of virulence (Figure 6c; Table 3). Similar trends were apparent when abundance was analyzed (Figure 6d; Table 3).

**Table 3:** Counts representing 14 OTUs assigned to the *Candidatus sulcia* clade together with the proportion of total reads represented by each taxon at generation 20 and corresponding *F*‐values (natal host and origin) for each OTU (univariate [abundance] and multivariate [proportions] GLMs; see also Figure 5c,d)

*Candidatus* OTUs	Numbers of samples with OTU	Total abundance	Abundance *F*‐natal†	Abundance *F*‐origin†	Proportion of total	Proportion *F*‐natal^a^^,^ †	Proportion *F*‐origin^a^^,^ †
OTU_1	20	478,499	6.945**	11.119***	0.530	4.264*	126.126***
OTU_8	20	78,192	4.251*	50.471***	0.087	8.040**	100.767***
OTU_10	20	106,135	5.806**	45.745***	0.118	9.543**	67.208***
OTU_12	20	82,806	7.720**	38.548***	0.092	8.244**	45.566***
OTU_14	20	2,066	2.518ns	7.653***	0.002	1.694ns	2.928ns
OTU_16	20	6,585	4.544*	20.560***	0.007	2.356ns	44.168***
OTU_17	20	127,839	6.197**	20.273***	0.142	5.352**	47.100***
OTU_22	20	3,059	4.849**	5.160**	0.003	3.710*	49.362***
OTU_29	20	1,807	5.143*	14.336***	0.002	1.451ns	5.649**
OTU_31	19	659	6.668**	16.880***	0.001	5.988**	24.817***
OTU_37	20	1,945	3.676*	26.649***	0.002	6.846**	4.371*
OTU_46	13	138	1.489ns	0.935ns	0.000	2.185ns	0.991ns
OTU_54	20	12,324	0.534ns	11.453***	0.014	0.948ns	8.068**
OTU_58	11	437	0.171ns	0.528ns	<0.001	0.827ns	0.560ns

a Based on multivariate GLM.

^†^ns = *p* ≥ .05, *p ≤ .05, **p ≤ .01, ***p ≤ .001; DF natal host = 3, 12, DF origin = 4, 12.

### 3.6 Leafhopper microbiome after phase II selection

There were significant differences in the leafhopper microbiome between origins at the OTU level (Pseudo‐*F* = 5.1337, *p* = .005), as shown in the MDS plot (Figure 5c). Pairwise tests showed statistically significant differences between San Pablo and every other collection site (all with *p*‐values ≤ .05), as well as between Batangas and Laguna (*t* = 3.177, *p* = .031). These differences between origins were mostly attributed to five OTUs (no. 1, 8, 10, 12, 17) assigned to the *Candidatus sulcia* clade (Table S7).

Similar results were observed at the genus level, in which “origin” had a significant impact on the microbiome community (Pseudo‐*F* = 5.487, *p* = .001; Figure 5d). Pairwise differences were found between San Pablo and every other collection site (all *p*‐values ≤ .05), as well as between Batangas and Laguna (*t* = 3.042, *p* = .019) and Batangas and Quezon (*t* = 2.799, *p* = .028). Several taxa contributed to these differences, particularly *Paenirhodobacter* spp., *Streptococcus* spp., *Gluconobacter* spp., and other unclassified taxa (Table S8). Significant PERMANOVA results in phase II were not affected by data dispersion, as confirmed by PERMDISP tests, which all showed nonsignificant *p*‐values (>.05).

### 3.7 Changes in abundance of key taxa during phase II selection

**Figure 8:**
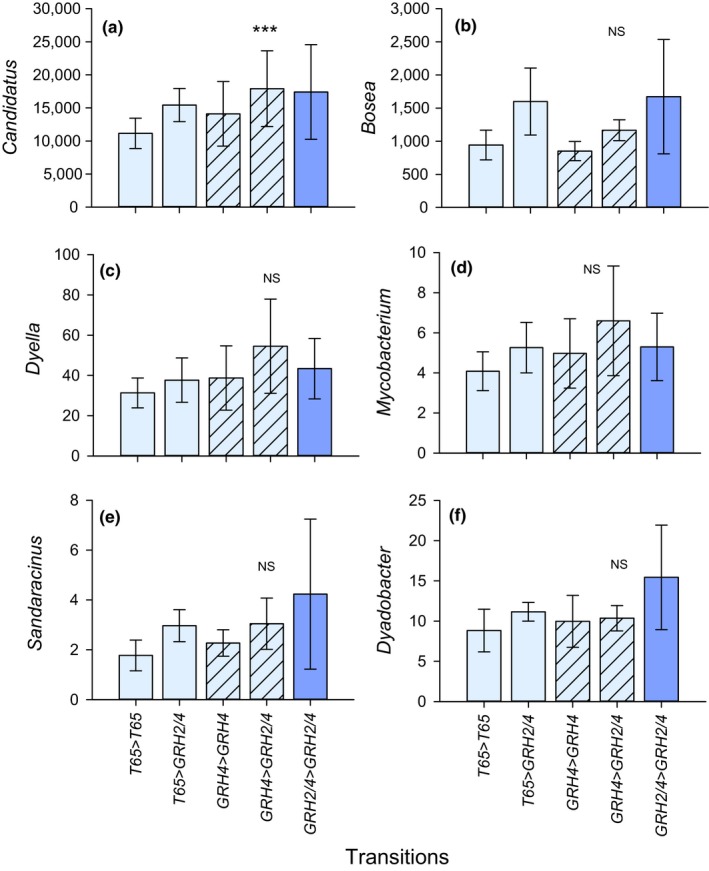
The relative abundance of key genera associated with the microbiomes of leafhoppers reared for 20 generations on T65 (light shading) or *GRH4*‐NIL (hatched) and either maintained on the same natal host or switched to *GRH2/GRH4*‐PYL for six generations. The abundances on leafhoppers that were maintained on *GRH2/GRH4*‐PYL (dark shading) for 26 generations are included for comparison. The genera are from the (a) *Candidatus sulcus* clade, (b) *Bosea*, (c) *Dyella*, (d) *Mycobacterium*, (e) *Sandaracinus*, and (f) *Dyadobacter*. Error bars are indicated. NS = no significant effect of transition, ***statistically significant effect (*p* ≤ .001, *N* = 5 colonies)

OTUs assigned to the *Candidatus sulcia* clade were not affected by initial leafhopper natal host (T65 or *GRH4*‐NIL; *F*_1,16_ < 0.001, *p* = 1.000; ranked data), but gained prominence in leafhoppers after six generations on *GRH2/GRH4*‐PYL (*F*_1,16_ = 10.889, *p* = .005; ranked data). The interaction term was not significant (Figure 8a). Although patterns were often consistent, *Bosea*, *Dyadobacter*, *Dyella*, *Mycobacterium*, and *Sandaracinus* were not statistically significantly affected by the initial natal hosts of leafhoppers or by switching the leafhoppers to *GRH2/GRH4*‐PYL for six generations (univariate GLMs, all *p*‐values > 0.05; Figure 8b–f).

## 4 DISCUSSION

Our results depict a highly diverse leafhopper microbiome that in virulence adapted colonies were influenced more by population origin than by exposure to resistance genes. However, six bacterial taxa, including the obligate symbiont *Candidatus sulcia* clade, showed abundance patterns that reflected the virulence of the leafhopper hosts on *GRH2/GRH4*‐PYL (after phase I selection). These patterns were consistent during a second selection phase when their insect hosts were switched from T65 or *GRH4*‐NIL to *GRH2/GRH4*‐PYL for six generations (phase II selection).

### 4.1 The leafhopper microbiome—diversity and function

DNA sequencing of 16s rRNA‐V5 amplicons yielded 227 OTUs from the 45 leafhopper samples. These were assigned to 72 taxa (and a further unclassified group) based on NCBI Blasting. The number of OTUs per sample varied between 69 and 119, which is within the range of OTU richness recorded from similar studies for other plant–herbivore systems (e.g., Alonso‐Pernas et al., 2017; Grünwald, Pilhofer, & Höll, 2010; Montagna et al., 2015; Suen et al., 2010; Tang et al., 2010). Our PERMANOVA analyses indicated that, when all OTUs or genera were included together, the origin of the leafhopper colonies was a more significant determinant of microbiome community structure than was the host plant and its resistance genes. A similar observation was made when the OTU composition of *Candidatus sulcia* was compared across colonies and host plants (Figure 5). The similarity of microbiomes among colonies that were derived from the same initial populations (i.e., either Batangas, Laguna, Quezon, Rizal, San Pablo), irrespective of host plant, is noteworthy because colonies had been selected for 20–26 generations (>30 months) without appreciable divergence in the microbiome compositions among derived colonies. This occurred despite maintaining colonies under relatively nonsterile conditions (using heat‐treated soil in unsterilized pots and without any periodic sterilization of cages). We surface‐sterilized our samples before analyses to remove exogenous bacteria; however, we are unaware of the effectiveness of this for leafhoppers and assume that exogenous bacterial DNA was still associated with the insect integument. Hammer, Dickerson, and Fierer (2015) demonstrated that surface sterilization of insects does not appreciably change bacterial community structure compared to unsterilized insects, suggesting either that the vast majority of bacteria occur inside the insect body, or that surface sterilization has little effect on exogenous bacterial DNA. In our study, the relatively large effect of collection site (origin) on microbiome composition suggests that much of the bacteria in the microbiome include taxa picked up as the leafhoppers move through their environment. It further suggests that much of the microbiome is transmitted vertically from parents to offspring either through the egg (Kobialka, Michalik, Walczak, Junkiert, & Szklarzewicz, 2016; Michalik, Jankowska, Kot, Golas, & Szklarzewicz, 2014) or because these shared common feeding sites (see Ferrater & Horgan, 2016). However, because leafhoppers were continuously caged in our experiments without possibilities for long‐distance movement, it is possible that the microbiomes remained more stable than would occur in wild populations.

This diverse community of bacteria in the leafhopper microbiome may contribute in several ways to the insect herbivores. The community also included endophyte bacteria (Gadhave & Gange, 2016). Some of the endophyte bacteria that we detected could function in plant protection against insects and diseases, including leafhoppers, representing an important function of the plant microbiome that interacts with the leafhopper populations; however, bacterial function is difficult to determine from 16S sequencing. For example, bacteria of the genus *Serratia* were detected among colonies derived from all five original locations (Table S3). The bacterium *Serratia marcescens* is pathogenic to *N. lugens* (Niu, Liu, Li, & Guo, 2016). In contrast, certain *Bacillus* spp. (*B. amyloliquefaciens*, *B. pumilus*, and *B. subtilis*) that occur on the integument of rice planthoppers can inhibit the effects of pathogenic microbes such as the entomopathogenic fungi *Beauveria bassiana* and *Metarhizium anisopliae* (Toledo, Lopez, Aulicino, Lenicov, & Balatti, 2015). The herbivore microbiome may also include species that are pathogenic to rice. For example, we detected bacteria of the genus *Xanthomonas* present at low levels in all leafhopper colonies. *Xanthomonas oryzae* is a considerable pathogen of rice and has been shown to result in complex interactions with insect herbivores (*N. lugens*) and their predators (*Cyrtorhinus lividipennis*), albeit in experiments with highly diseased plants (Sun et al., 2016). Carrying such pathogens on the integument could ultimately benefit leafhoppers by inducing host susceptibility (Chung et al., 2013, and see below). Plant viruses, such as the rice dwarf virus that is vectored by leafhoppers, have recently been shown to attach to the envelope of *Candidatus sulcia* where they are transmitted to leafhopper eggs (Jia et al., 2017). We did not detect *Wolbachia* in our experiments; however, it has been suggested that *Wolbachia* are eliminated from insect colonies that have been reared for several generations under controlled conditions (Wang et al., 2015, 2016).

### 4.2 Endosymbionts associated with leafhopper feeding and virulence adaptation

There is considerable evidence that bacteria and fungi that are facultatively or obligately symbiotic with rice planthoppers and leafhoppers are essential for normal insect development. These often ancient mutualisms have resulted in some symbiotic bacteria, including members of the *Candidatus sulcia* clade, losing key functional genes that would otherwise allow them to exist independently of the host insect (Bennett, McCutcheon, MacDonald, Romanovicz, & Moran, 2014). Unlike planthoppers that require YLS for nitrogen metabolism, the *N. virescens* in our colonies did not possess YSL (J. B. Ferrater, unpublished data). However, they did harbor high densities of obligate symbiont bacteria assigned to the *Candidatus sulcia* clade. We did not identify these bacteria, but they possibly include strains of *Candidatus sulcia muelleri* that occur in the closely related *N. cincticeps* (Noda et al., 2012). These bacteria occur in bacteriocytes within the large bacteriomes of *N. cincticeps*. Noda et al. (2012) indicated that the bacteriomes include two types of bacteriocyte that each harbored different symbiotic bacteria: *Candidatus sulcia muelleri* in outer regions, and a further β‐proteobacterial symbiont in the inner region of the bacteriomes. A further symbiont assigned to the α‐proteobacterial genus *Rikettsia* also occurs in *N. cincticeps* (Noda et al., 2012). As with YLS (Nan et al., 2016), symbiotic bacteria from the bacteriomes are transovarially transmitted from parents to offspring (Kobialka et al., 2016; Michalik et al., 2014). Furthermore, *Candidatus sulcia* likely plays a role in host nutrition; for example, *Candidatus sulcia muelleri* can synthesize essential amino acids (Bennett & Moran, 2013) and a recent genomic analysis of the closely related *Candidatus arsenophorus nilaparvatae*, which occurs in rice planthoppers, suggests that the bacterium plays a role in B vitamin synthesis (Fan, Lu, Ye, Yu, & Zhang, 2016).

In contrast to the often strong evidence that symbionts play a role in host nutrition, evidence for their role in virulence adaptation has been inconclusive. This is largely due to a lack of agreement between the results from similar experiments conducted by different research groups (e.g., YLS: Lu et al., 2004 and Ferrater et al., 2015; Lu et al., 2004 and Wang et al., 2015; Chen et al., 2011 and Horgan & Ferrater, 2017; endosymbiotic bacteria: Xu et al., 2015 and Wang et al., 2015). For example, although *Chryseobacterium* (Bacteroidetes), *Acinetobacter*, *Arsenophonus*, *Arthrobacter*, and *Serratia* (all Gammaproteobacteria) featured prominently in the microbiomes of laboratory planthopper colonies, patterns in the abundance of these bacterial taxa from planthoppers selectively reared on three natal rice plants (TN1, Mudgo, or ASD7) were different in studies by Xu et al. (2015) and Wang et al. (2015).

In our study, six bacterial taxa showed positive associations with the virulence of selected leafhopper colonies on the highly resistance *GRH2/GRH4*‐PYL. This included colonies selected on the monogenic NILs but capable of feeding on the pyramided line. The taxa were assigned to the obligate symbiont *Candidatus sulcia* clade, *Bosea*, *Dyella*, *Mycobacterium*, and *Sandaracinus*, each of which occurred at lowest densities on the susceptible T65, as well as *Dyanobacter*, which made a lower proportional contribution to the microbiome of T65 compared to the microbiomes associated with resistant lines (Table 2). Predictably higher densities from leafhoppers switched from T65 to *GRH2/GRH4*‐PYL further suggested that these taxa play a role in leafhopper nutrition. Although the results from phase II were generally weak (only the results for *Candidatus sulcia* were statistically significant), nevertheless the patterns were consistent between both selection phases (I and II). Relatively weak responses during phase II selection may be due to the short selection period (six generations), which only allows partial adaptation to the novel host (Horgan et al., 2018; Rapusas & Heinrichs, 1990; Vu et al., 2014). Five of the endosymbiont taxa in particular (*Bosea*, *Candidatus sulcia*, *Dyella*, *Mycobacterium*, and *Sandaracinus*) showed strong responses to phase I selection and consistent response patterns from phase II selection. Bacteria of the genus *Bosea*, which occurred at high densities in our leafhopper samples, have been linked to nitrogen metabolism (denitrification in soil) and occur in the hind wall of the cockchafer gut (Alonso‐Pernas et al., 2017; Dandie et al., 2008). *Bosea* have also been associated with detoxification of bendiocarb and carbofuran insecticides (Jiménez‐Arévalo, Ahuatzi‐Chacón, Galíndez‐Mayer, Juárez‐Ramírez, & Ruiz‐Ordaz, 2016; Shin, Kim, Seong, Song, & Ka, 2012). *Sandaracinus* bacteria have been associated with the degradation of complex molecules including starch (Mohr, Garcia, Gerth, Irschik, & Müller, 2012; Sharma, Khatri, & Subramanian, 2016) and in sterol synthesis (Wei, Yin, & Welander, 2016). Bacteria of the genus *Dyella* have been recorded as endophytes associated with rice seeds (Hardoim, Hardoim, Overbeek, & Elsas, 2012). *Dyella*‐like bacteria are linked to a diversity of ecological functions including the breakdown of starch (Anandham et al., 2008) and antagonistic effects on plant pathogens (Iasur‐Kruh et al., 2018). Ferrater and Horgan (2016) suggested that unknown factors that may include endosymbiont bacteria are transmitted between virulent and avirulent planthoppers at shared feeding sites and can increase the feeding success of avirulent individuals. Furthermore, this improved virulence was passed to successive planthopper generations (Ferrater & Horgan, 2016). Such bacteria may act as “decoys” that shift the target of induced host plant responses from herbivores to pathogens (Chung et al., 2013). Consistent trends in our results after phase I selection and the predictable changes during phase II selection suggest that the six taxa are associated with leafhopper feeding physiology; however, there is still no clear evidence that they determine virulence and virulence mechanisms remain to be elucidated.

### 4.3 Mechanisms of leafhopper virulence adaptation and future research

Compared to planthoppers, leafhopper virulence adaptation to resistant hosts has received relatively little research attention. Evidence suggests that leafhoppers can adapt to feed on resistant rice plants within five to ten generations of selection, but that they adapt more slowly (10–20 generations) to lay eggs on resistant varieties (Heinrichs & Rapusas, 1985; Horgan et al., 2018; Rapusas & Heinrichs, 1990). This suggests that the mechanisms that underlie feeding adaptation are different from those underlying adaptation to egg laying. Asano et al. (2015) indicated that resistance to leafhoppers in *GRH2/GRH4*‐PYL is associated with genes for proteinase inhibitors and cytochrome P450s. Attacks by *N. cincticeps* on *GRH2/GRH4*‐PYL also produced a strong induction of sesquiterpene volatiles (Asano et al., 2015). Virulence adaptation might therefore include short‐term (within a few generations) desensitizing to deterrent volatiles that allows feeding on resistant lines during a slower build‐up (up to 20 generations) of individuals capable of laying eggs on the resistant varieties. Microbes might respond to poor nutrient environments through community or population (strain) selection toward novel resource assimilation capacities. Bacteria may also play a role in detoxifying secondary plant defenses. For example, Malathi et al. (2018) found that bacterial communities associated with insecticide‐resistant *N. lugens* were enriched with bacteria involved in detoxification (see also Jiménez‐Arévalo et al., 2016; Shin et al., 2012). Alyokhin and Chen (2017) suggest that such bacteria may also determine the virulence of herbivores on resistant crops through detoxification of secondary chemicals involved in plant defenses. Despite the feasibility of these mechanisms, evidence for their role in virulence adaptation remains elusive.

## CONFLICT OF INTEREST

None declared.

## AUTHOR CONTRIBUTIONS

FGH, TSS, and RO designed the study; FGH, TSS, CCB, MLPA, and AFR performed the research; FGH, TSS, EC‐M, RO, and ILQ analyzed the data; FGH and TSS wrote the manuscript.

## Data Availability

Original data have been deposited in Dryad: https://doi.org/10.5061/dryad.td3fb01.
