# Supplementary material for: Microbiome responses during virulence adaptation by a phloem‐feeding insect to resistant near‐isogenic rice lines
Source: Ecol Evol. 2019 Oct 4;9(20):11911–29. doi: 10.1002/ece3.5699 (PMC6822046; doi:10.1002/ece3.5699)
Supplement: Supplementary file 1 [file ECE3-9-11911-s001.docx]

**Supplementary information - Table S1** Results of bioassays conducted on leafhopper colonies (A) prior to selection and (B, C) after 20 generations of selection. Leafhoppers were tested for virulence against (A, B) their natal hosts and (C) the pyramided resistant line *GRH2/GRH4-*PYL

| Rice line | Proportion of nymphs surviving^†^ | Biomass of surviving nymphs (mg d wgt)^†^ | Proportion developing to adult^†^ | Proportion of adults surviving^†^ | Biomass of surviving adults (mg d wgt)^†^ | Number of eggs laid per plant^†^ |
| --- | --- | --- | --- | --- | --- | --- |
| A: Pre-selection populations on natal hosts | | |  |  |  |  |
| T65 | 0.83±0.04b | 5.72±0.19c | 0.34±0.08bc | 0.83±0.02b | 4.99±0.07b | 40.40±11.22b |
| *GRH2*-NIL | 0.85±0.03b | 6.02±0.14c | 0.42±0.05c | 0.79±0.02b | 4.34±0.34b | 45.10±9.40b |
| *GRH4*-NIL | 0.83±0.06b | 4.69±0.37b | 0.18±0.04ab | 0.79±0.05b | 4.91±0.44b | 49.10±10.99b |
| *GRH2/GRH4*-PYL | 0.14±0.06a | 0.21±0.09a | 0.00±0.00a | 0.27±0.03a | 1.19±0.18a | 23.67±8.03a |
| F-host^‡^ | 109.261*** | 284.785*** | 11.982*** | 73.681*** | 37.425*** | 15.817*** |
| F-colony^‡^ | 4.766* | 4.897* |  |  |  | 47.561*** |
| B: Generation 20 populations on natal hosts | | |  |  |  |  |
| T65 | 0.90±0.04 | 2.48±0.40 | 0.00±0.00 | 0.80±0.15 | 6.08±1.30 | 48.60±6.99 |
| *GRH2*-NIL | 0.88±0.06 | 2.96±0.71 | 0.08±0.04 | 0.88±0.05 | 6.01±0.84 | 36.40±5.08 |
| *GRH4*-NIL | 0.98±0.02 | 3.11±0.30 | 0.06±0.06 | 0.80±0.11 | 5.70±0.81 | 28.20±9.49 |
| *GRH2/GRH4*-PYL | 0.96±0.02 | 4.53±0.35 | 0.13±0.09 | 0.80±0.13 | 5.62±1.06 | 53.40±11.20 |
| F-host^‡^ | 1.095ns | 2.933ns | 1.347ns | 0.039ns | 0.050ns | 2.110ns |
| C: Generation 20 populations on GRH2/GRH4-PYL | | |  |  |  |  |
| T65 | 0.94±0.02 | 1.46±0.15a | 0.00±0.00 | 0.76±0.12 | 5.08±1.08 | 22.60±3.53a |
| *GRH2*-NIL | 0.98±0.00 | 3.22±0.70ab | 0.00±0.00 | 0.80±0.09 | 5.39±0.56 | 34.20±2.20b |
| *GRH4*-NIL | 0.96±0.00 | 3.88±0.72b | 0.00±0.00 | 0.80±0.11 | 6.29±1.21 | 36.00±3.18b |
| *GRH2/GRH4*-PYL | 1.00±0.00 | 3.64±0.48b | 0.00±0.00 | 0.84±0.08 | 6.80±0.73 | 35.60±5.10b |
| F-host^‡^ | 0.975ns | 3.878* | - | 0.0.39ns | 0.723ns | 3.469* |

†: Numbers are averages ± SEM (N = 5 colonies); lowercase letters indicate homogenous groups.

‡: F-host for univariate GLM, DF = 3,16 where colony (block) was not significant; F-host DF = 3,12 where the factor colony was included; F-colony DF = 3,12; ns = P ≥ 0.05, * = P ≤ 0.05, *** = P ≤ 0.005. Survival data were log+1 transformed and proportions arcsine transformed before analysis.

**Table S2** Results of repeated measures GLM of virulence bioassays during the six generations of phase II selection (see also Fig. 4)

| Sources of variation | DF | F-values^†^ |  |  |  |  |  |  |
| --- | --- | --- | --- | --- | --- | --- | --- | --- |
|  |  | Adult survival | Adult biomass | Eggs laid | Nymph survival | Nymph biomass | Nymph development | Plant biomass |
| *Within subject effects* |  |  |  |  |  |  |  |  |
| Generation | 5 | 17.554*** | 9.991*** | 16.928*** | 11.053*** | 49.909*** | 39.580*** | 124.966*** |
| Linear contrast | 1 | 67.268*** | 25.940*** | 153.470*** | 35.908*** | 12.261** | 0.109ns | 9.359** |
| Generation × rice line | 15 | 0.590ns | 1.044ns | 1.100ns | 1.734ns | 1.361ns | 2.327** | 1.397ns |
| Error | 80 |  |  |  |  |  |  |  |
| *Between subject effects* |  |  |  |  |  |  |  |  |
| Rice line | 3 | 0.487ns | 0.283ns | 0.102ns | 2.245ns | 3.974* | 5.920** | 5.183** |
| Error | 16 |  |  |  |  |  |  |  |

†: ns = P ≤ 0.05, * = P ≥ 0.05, ** = P ≤ 0.01, *** = P ≤ 0.005; data for survival were arcsine transformed, biomass was log+1 transformed and nymph development (the proportion reaching the adult stage) was ranked prior to analyses.

**Table S3** Summary of OTUs based on 45 leafhopper colony samples sequenced for the 16S rRNA gene

| Phylum | Genus | Number of OTUs | Phase I selection |  |  |  | Phase II selection |  |  |  | Both phases |  |
| --- | --- | --- | --- | --- | --- | --- | --- | --- | --- | --- | --- | --- |
|  |  |  | Number of sites | Number of NILs | Total reads | Proportion of samples | Number of sites | Number of transitions | Total reads | Proportion of samples | Number of reads | Proportion of samples |
| Actinobacteria | |  |  |  |  |  |  |  |  |  |  |  |
|  | *Conexibacter* | 1 | 5 | 4 | 37 | 0.50 | 1 | 1 | 1 | 0.04 | 38 | 0.24 |
|  | *Curtobacterium* | 1 | 4 | 4 | 5867 | 0.65 | 5 | 5 | 39 | 0.40 | 5899 | 0.51 |
|  | *Mycobacterium* | 1 | 5 | 4 | 367 | 0.95 | 5 | 5 | 608 | 1.00 | 971 | 0.98 |
|  | *Nocardioides* | 1 | 1 | 2 | 8 | 0.10 | 0 | 0 | 0 | 0.00 | 8 | 0.04 |
| Bacteroidetes | |  |  |  |  |  |  |  |  |  |  |  |
|  | *Asinibacterium* | 1 | 2 | 3 | 7 | 0.15 | 1 | 1 | 2 | 0.04 | 9 | 0.09 |
|  | *Candidatus sulcia* | 28 | 5 | 4 | 890517 | 1.00 | 5 | 5 | 1742571 | 1.00 | 2609988 | 1.00 |
|  | *Dyadobacter* | 1 | 5 | 4 | 568 | 0.95 | 5 | 5 | 1330 | 1.00 | 1874 | 0.98 |
|  | *Fluviicola* | 1 | 5 | 4 | 40 | 0.70 | 5 | 5 | 94 | 0.76 | 133 | 0.73 |
|  | *Portibacter* | 1 | 4 | 4 | 15 | 0.30 | 4 | 4 | 8 | 0.20 | 23 | 0.24 |
|  | *Runella* | 1 | 2 | 2 | 10 | 0.10 | 2 | 2 | 17 | 0.08 | 27 | 0.09 |
| Cyanobacteria | |  |  |  |  |  |  |  |  |  |  |  |
|  | unidentified | 3 | 4 | 4 | 39 | 0.50 | 5 | 5 | 26 | 0.48 | 65 | 0.49 |
| Firmicutes | |  |  |  |  |  |  |  |  |  |  |  |
|  | *Aeribacillus* | 1 | 4 | 3 | 9 | 0.25 | 5 | 5 | 29 | 0.56 | 36 | 0.42 |
|  | *Ammoniphilus* | 1 | 5 | 4 | 104 | 0.55 | 5 | 5 | 239 | 0.48 | 342 | 0.51 |
|  | *Anaerococcus* | 1 | 3 | 3 | 4 | 0.20 | 4 | 3 | 11 | 0.24 | 15 | 0.22 |
|  | *Anoxybacillus* | 2 | 4 | 4 | 8020 | 0.60 | 5 | 5 | 206 | 0.60 | 8225 | 0.60 |
|  | *Bacillus* | 3 | 2 | 3 | 7 | 0.15 | 5 | 5 | 2747 | 0.56 | 2754 | 0.38 |
|  | *Exiguobacterium* | 1 | 0 | 0 | 0 | 0.00 | 3 | 5 | 22030 | 0.40 | 22030 | 0.22 |
|  | *Pontibacillus* | 1 | 0 | 0 | 0 | 0.00 | 1 | 1 | 5 | 0.04 | 5 | 0.02 |
|  | *Salinicoccus* | 1 | 0 | 0 | 0 | 0.00 | 1 | 2 | 6 | 0.08 | 6 | 0.04 |
|  | *Staphylococcus* | 7 | 5 | 4 | 27234 | 1.00 | 5 | 5 | 27656 | 1.00 | 54011 | 1.00 |
|  | *Streptococcus* | 7 | 5 | 4 | 59824 | 1.00 | 5 | 5 | 56321 | 0.96 | 116140 | 0.98 |
|  | *Tenuibacillus* | 2 | 5 | 4 | 17197 | 1.00 | 5 | 5 | 101 | 0.96 | 17294 | 0.98 |
| Proteobacteria | |  |  |  |  |  |  |  |  |  |  |  |
|  | *Acinetobacter* | 1 | 5 | 4 | 42 | 0.75 | 5 | 5 | 24 | 0.60 | 66 | 0.67 |
|  | *Aeromonas* | 1 | 4 | 4 | 16327 | 0.55 | 2 | 4 | 10 | 0.16 | 16336 | 0.33 |
|  | *Ameyamaea* | 1 | 1 | 1 | 1 | 0.05 | 3 | 3 | 5 | 0.12 | 6 | 0.09 |
|  | *Azohydromonas* | 1 | 5 | 4 | 27 | 0.50 | 4 | 3 | 19 | 0.24 | 46 | 0.36 |
|  | *Azospira* | 1 | 1 | 1 | 3 | 0.05 | 5 | 4 | 16 | 0.32 | 19 | 0.20 |
|  | *Bartonella* | 1 | 5 | 4 | 328 | 0.80 | 5 | 5 | 394 | 0.60 | 721 | 0.69 |
|  | *Bdellovibrio* | 1 | 5 | 4 | 4754 | 0.65 | 5 | 4 | 35 | 0.36 | 4786 | 0.49 |
|  | *Bordetella* | 1 | 3 | 4 | 81 | 0.55 | 4 | 5 | 143 | 0.28 | 201 | 0.40 |
|  | *Bosea* | 1 | 5 | 4 | 73523 | 1.00 | 5 | 5 | 147271 | 1.00 | 212571 | 1.00 |
|  | *Bradyrhizobium* | 2 | 5 | 4 | 42310 | 1.00 | 5 | 5 | 41345 | 1.00 | 81039 | 1.00 |
|  | *Brevundimonas* | 1 | 4 | 4 | 6 | 0.25 | 0 | 0 | 0 | 0.00 | 6 | 0.11 |
|  | *Burkholderia* | 1 | 5 | 4 | 98 | 0.85 | 5 | 5 | 53 | 0.60 | 151 | 0.71 |
|  | *Caulobacter* | 1 | 5 | 4 | 98 | 0.65 | 1 | 4 | 9 | 0.16 | 105 | 0.38 |
|  | *Cedecea* | 6 | 5 | 4 | 44295 | 0.95 | 5 | 5 | 12790 | 0.88 | 57081 | 0.91 |
|  | *Comamonas* | 1 | 2 | 2 | 2 | 0.10 | 3 | 3 | 6 | 0.12 | 8 | 0.11 |
|  | *Dasania* | 1 | 0 | 0 | 0 | 0.00 | 1 | 1 | 3 | 0.04 | 3 | 0.02 |
|  | *Delftia* | 1 | 0 | 0 | 0 | 0.00 | 1 | 1 | 11 | 0.04 | 11 | 0.02 |
|  | *Diplorickettsia* | 9 | 5 | 4 | 47984 | 1.00 | 5 | 5 | 28420 | 0.96 | 76233 | 0.98 |
|  | *Dyella* | 1 | 5 | 4 | 2460 | 1.00 | 5 | 5 | 4690 | 1.00 | 7128 | 1.00 |
|  | *Gemmobacter* | 1 | 2 | 2 | 2 | 0.10 | 5 | 5 | 126 | 0.60 | 128 | 0.38 |
|  | *Georgfuchsia* | 1 | 4 | 4 | 74 | 0.60 | 4 | 4 | 28 | 0.36 | 102 | 0.47 |
|  | *Gluconobacter* | 11 | 5 | 4 | 144340 | 1.00 | 5 | 5 | 360897 | 1.00 | 504422 | 1.00 |
|  | *Haemophilus* | 5 | 5 | 4 | 106 | 0.90 | 5 | 5 | 199 | 0.96 | 302 | 0.93 |
|  | *Halomonas* | 2 | 3 | 2 | 3 | 0.15 | 4 | 4 | 13 | 0.32 | 16 | 0.24 |
|  | *Hydrogenophilus* | 3 | 5 | 4 | 13 | 0.35 | 5 | 5 | 112 | 0.80 | 124 | 0.60 |
|  | *Hyphomicrobium* | 1 | 5 | 4 | 26 | 0.60 | 5 | 5 | 41 | 0.76 | 66 | 0.69 |
|  | *Kingella* | 7 | 5 | 4 | 149975 | 1.00 | 5 | 5 | 144639 | 1.00 | 292365 | 1.00 |
|  | *Klebsiella* | 2 | 5 | 4 | 30 | 0.55 | 4 | 5 | 52154 | 0.64 | 52182 | 0.60 |
|  | *Lentilitoribacter* | 1 | 5 | 4 | 56 | 0.40 | 4 | 4 | 13 | 0.24 | 69 | 0.31 |
|  | *Limnobacter* | 1 | 3 | 3 | 23 | 0.25 | 5 | 5 | 21 | 0.32 | 44 | 0.29 |
|  | *Limnohabitans* | 1 | 4 | 4 | 122 | 0.45 | 5 | 5 | 42 | 0.56 | 164 | 0.51 |
|  | *Mesorhizobium* | 1 | 0 | 0 | 0 | 0.00 | 1 | 1 | 25 | 0.04 | 25 | 0.02 |
|  | *Methylobacillus* | 2 | 5 | 4 | 24494 | 0.70 | 4 | 5 | 31 | 0.36 | 24521 | 0.51 |
|  | *Methylotenera* | 1 | 5 | 4 | 2063 | 1.00 | 5 | 5 | 424 | 1.00 | 2472 | 1.00 |
|  | *Neisseria* | 14 | 5 | 4 | 259284 | 1.00 | 5 | 5 | 141915 | 1.00 | 396674 | 1.00 |
|  | *Nevskia* | 2 | 4 | 3 | 21 | 0.30 | 5 | 5 | 1061 | 0.84 | 1082 | 0.60 |
|  | *Paenirhodobacter* | 7 | 5 | 4 | 80678 | 0.90 | 4 | 5 | 115486 | 0.48 | 177580 | 0.67 |
|  | *Pantoea* | 1 | 1 | 1 | 1 | 0.05 | 5 | 3 | 15 | 0.32 | 16 | 0.20 |
|  | *Piscinibacter* | 2 | 5 | 4 | 4379 | 0.80 | 5 | 5 | 22 | 0.52 | 4400 | 0.64 |
|  | *Pseudomonas* | 6 | 5 | 4 | 4753 | 1.00 | 5 | 5 | 1233 | 1.00 | 5945 | 1.00 |
|  | *Reyranella* | 1 | 3 | 4 | 568 | 0.40 | 1 | 4 | 958 | 0.16 | 1371 | 0.27 |
|  | *Rhodoferax* | 1 | 0 | 0 | 0 | 0.00 | 2 | 3 | 9 | 0.16 | 9 | 0.09 |
|  | *Rubrivivax* | 1 | 5 | 4 | 46 | 0.55 | 5 | 5 | 36 | 0.60 | 82 | 0.58 |
|  | *Sandaracinus* | 1 | 5 | 4 | 171 | 0.90 | 5 | 5 | 308 | 1.00 | 473 | 0.96 |
|  | *Serratia* | 1 | 4 | 3 | 51 | 0.20 | 3 | 4 | 13 | 0.32 | 64 | 0.27 |
|  | *Sphingomonas* | 1 | 5 | 4 | 85 | 0.75 | 5 | 5 | 199 | 0.96 | 283 | 0.87 |
|  | *Tepidiphilus* | 1 | 5 | 4 | 1943 | 1.00 | 5 | 5 | 1780 | 1.00 | 3686 | 1.00 |
|  | *Thiovirga* | 1 | 3 | 3 | 7 | 0.25 | 1 | 1 | 2 | 0.04 | 9 | 0.13 |
|  | *Xanthomonas* | 1 | 5 | 4 | 690 | 0.75 | 1 | 4 | 15 | 0.16 | 702 | 0.42 |
|  | *Zavarzinia* | 1 | 0 | 0 | 0 | 0.00 | 1 | 1 | 5 | 0.04 | 5 | 0.02 |
| Unclassified | | 45 | 5 | 4 | 206666 | 1.00 | 5 | 5 | 404255 | 1.00 | 561733 | 1.00 |
| Total reads | |  |  |  | 2122883 |  |  |  | 3315368 |  | 5327526 |  |
| Total OTUs | | 227 |  |  |  |  |  |  |  |  |  |  |
| Total genera | | 73 |  |  |  |  |  |  |  |  |  |  |

**Table S4** SIMPER analysis of leafhopper microbial communities (phase I) showing cumulative percentages of dissimilarity (based on average square-rooted abundance of OTUs) between significantly different pairs of the factor ‘location’.

| Groups Batangas & Quezon | |  |  |  |  |
| --- | --- | --- | --- | --- | --- |
| Average dissimilarity = 29.17 | |  |  |  |  |
|  |  |  |  |  |  |
|  | Group Batangas | Group Quezon |  |  |  |
| Species | Av.Abund | Av.Abund | Diss/SD | Contrib% | Cum.% |
| OTU_1 | 112.56 | 184.24 | 2.89 | 26.07 | 26.07 |
| OTU_17 | 61.72 | 106.59 | 3.49 | 16.21 | 42.28 |
| OTU_54 | 38.28 | 1.67 | 5.15 | 13.05 | 55.33 |
| OTU_10 | 43.22 | 78.45 | 4.41 | 12.67 | 68 |
| OTU_8 | 34.82 | 69.53 | 4.56 | 12.47 | 80.47 |
|  |  |  |  |  |  |
| Groups Laguna & Quezon | |  |  |  |  |
| Average dissimilarity = 28.01 | |  |  |  |  |
|  |  |  |  |  |  |
|  | Group Laguna | Group Quezon |  |  |  |
| Species | Av.Abund | Av.Abund | Diss/SD | Contrib% | Cum.% |
| OTU_1 | 102.76 | 184.24 | 3 | 30.06 | 30.06 |
| OTU_17 | 53.14 | 106.59 | 3.55 | 19.65 | 49.71 |
| OTU_54 | 38.51 | 1.67 | 3.17 | 13.52 | 63.23 |
| OTU_8 | 42.76 | 69.53 | 5.23 | 9.78 | 73.01 |
| OTU_10 | 53.4 | 78.45 | 5.19 | 9.17 | 82.18 |
|  |  |  |  |  |  |
| Groups Batangas & Rizal | |  |  |  |  |
| Average dissimilarity = 35.43 | |  |  |  |  |
|  |  |  |  |  |  |
|  | Group Batangas | Group Rizal |  |  |  |
| Species | Av.Abund | Av.Abund | Diss/SD | Contrib% | Cum.% |
| OTU_10 | 43.22 | 115.3 | 5.47 | 19.14 | 19.14 |
| OTU_1 | 112.56 | 182.35 | 2.83 | 18.37 | 37.52 |
| OTU_8 | 34.82 | 102.83 | 5.94 | 18.07 | 55.58 |
| OTU_12 | 44.37 | 96.98 | 4.14 | 14.05 | 69.63 |
| OTU_17 | 61.72 | 99.83 | 2.55 | 9.94 | 79.57 |
|  |  |  |  |  |  |
| Groups Laguna & Rizal | |  |  |  |  |
| Average dissimilarity = 34.43 | |  |  |  |  |
|  |  |  |  |  |  |
|  | Group Laguna | Group Rizal |  |  |  |
| Species | Av.Abund | Av.Abund | Diss/SD | Contrib% | Cum.% |
| OTU_1 | 102.76 | 182.35 | 2.84 | 21.08 | 21.08 |
| OTU_10 | 53.4 | 115.3 | 4.77 | 16.57 | 37.65 |
| OTU_8 | 42.76 | 102.83 | 5.78 | 16.1 | 53.75 |
| OTU_17 | 53.14 | 99.83 | 2.97 | 12.32 | 66.06 |
| OTU_12 | 53.9 | 96.98 | 3.43 | 11.53 | 77.59 |
|  |  |  |  |  |  |
| Groups Quezon & Rizal | |  |  |  |  |
| Average dissimilarity = 12.30 | |  |  |  |  |
|  |  |  |  |  |  |
|  | Group Quezon | Group Rizal |  |  |  |
| Species | Av.Abund | Av.Abund | Diss/SD | Contrib% | Cum.% |
| OTU_10 | 78.45 | 115.3 | 4.12 | 23.15 | 23.15 |
| OTU_8 | 69.53 | 102.83 | 4.46 | 20.94 | 44.09 |
| OTU_12 | 65.81 | 96.98 | 4.26 | 19.58 | 63.67 |
| OTU_1 | 184.24 | 182.35 | 2.06 | 12.35 | 76.02 |
|  |  |  |  |  |  |
| Groups Batangas & SanPablo | | |  |  |  |
| Average dissimilarity = 17.43 | |  |  |  |  |
|  |  |  |  |  |  |
|  | Group Batangas | Group SanPablo |  |  |  |
| Species | Av.Abund | Av.Abund | Diss/SD | Contrib% | Cum.% |
| OTU_1 | 112.56 | 159.32 | 1.99 | 34.19 | 34.19 |
| OTU_54 | 38.28 | 2.8 | 4.63 | 27.27 | 61.46 |
| OTU_17 | 61.72 | 55.07 | 2.7 | 7.66 | 69.12 |
| OTU_8 | 34.82 | 26.54 | 1.3 | 5.83 | 74.95 |
| OTU_12 | 44.37 | 39.15 | 2.69 | 5.59 | 80.55 |
|  |  |  |  |  |  |
| Groups Laguna & SanPablo | |  |  |  |  |
| Average dissimilarity = 22.26 | |  |  |  |  |
|  |  |  |  |  |  |
|  | Group Laguna | Group SanPablo |  |  |  |
| Species | Av.Abund | Av.Abund | Diss/SD | Contrib% | Cum.% |
| OTU_1 | 102.76 | 159.32 | 1.51 | 31.61 | 31.61 |
| OTU_54 | 38.51 | 2.8 | 3.71 | 20.86 | 52.47 |
| OTU_12 | 53.9 | 39.15 | 1.64 | 9.98 | 62.45 |
| OTU_8 | 42.76 | 26.54 | 2.17 | 9.55 | 72 |
| OTU_10 | 53.4 | 41.69 | 1.95 | 8.6 | 80.6 |
|  |  |  |  |  |  |
| Groups Quezon & SanPablo | |  |  |  |  |
| Average dissimilarity = 24.25 | |  |  |  |  |
|  |  |  |  |  |  |
|  | Group Quezon | Group SanPablo |  |  |  |
| Species | Av.Abund | Av.Abund | Diss/SD | Contrib% | Cum.% |
| OTU_17 | 106.59 | 55.07 | 3.94 | 22.72 | 22.72 |
| OTU_8 | 69.53 | 26.54 | 5.47 | 18.67 | 41.39 |
| OTU_10 | 78.45 | 41.69 | 3.08 | 16.3 | 57.69 |
| OTU_1 | 184.24 | 159.32 | 1.06 | 13.25 | 70.93 |
| OTU_12 | 65.81 | 39.15 | 2.41 | 11.97 | 82.9 |
|  |  |  |  |  |  |
| Groups Rizal & SanPablo | |  |  |  |  |
| Average dissimilarity = 31.01 | |  |  |  |  |
|  |  |  |  |  |  |
|  | Group Rizal | Group SanPablo |  |  |  |
| Species | Av.Abund | Av.Abund | Diss/SD | Contrib% | Cum.% |
| OTU_8 | 102.83 | 26.54 | 10.69 | 23.3 | 23.3 |
| OTU_10 | 115.3 | 41.69 | 6.01 | 22.71 | 46.01 |
| OTU_12 | 96.98 | 39.15 | 4.96 | 17.93 | 63.95 |
| OTU_17 | 99.83 | 55.07 | 3.23 | 13.63 | 77.57 |

**Table S5** SIMPER analysis of leafhopper microbial communities (phase I) showing cumulative percentages of dissimilarity (based on average square-rooted abundance of OTUs) between significantly different pairs of the factor ‘natal’.

| Groups GRH2 & T65 | |  |  |  |  |  |
| --- | --- | --- | --- | --- | --- | --- |
| Average dissimilarity = 15.29 | | |  |  |  |  |
|  |  |  |  |  |  |  |
|  | Group GRH2 | Group T65 |  |  |  |  |
| Species | Av.Abund | Av.Abund | Av.Diss | Diss/SD | Contrib% | Cum.% |
| OTU_1 | 151.29 | 110.35 | 4.98 | 2.76 | 32.56 | 32.56 |
| OTU_17 | 78.36 | 56.01 | 2.57 | 2.53 | 16.8 | 49.36 |
| OTU_10 | 64.54 | 51.74 | 1.59 | 2.85 | 10.4 | 59.75 |
| OTU_12 | 58.49 | 46.22 | 1.57 | 2.34 | 10.26 | 70.02 |
| OTU_8 | 53.77 | 44.22 | 1.11 | 1.69 | 7.27 | 77.29 |
|  |  |  |  |  |  |  |
| Groups GRH2/GRH4 & T65 | | |  |  |  |  |
| Average dissimilarity = 21.05 | | |  |  |  |  |
|  |  |  |  |  |  |  |
|  | Group GRH2/GRH4 | Group T65 |  |  |  |  |
| Species | Av.Abund | Av.Abund | Av.Diss | Diss/SD | Contrib% | Cum.% |
| OTU_1 | 159.21 | 110.35 | 5.96 | 1.85 | 28.3 | 28.3 |
| OTU_10 | 79.52 | 51.74 | 3.19 | 3.97 | 15.15 | 43.46 |
| OTU_12 | 72.38 | 46.22 | 3.06 | 2.85 | 14.56 | 58.01 |
| OTU_17 | 78.87 | 56.01 | 2.69 | 2.53 | 12.76 | 70.77 |
| OTU_8 | 65.63 | 44.22 | 2.37 | 3.42 | 11.27 | 82.04 |
|  |  |  |  |  |  |  |
| Groups GRH4 & T65 | |  |  |  |  |  |
| Average dissimilarity = 19.76 | | |  |  |  |  |
|  |  |  |  |  |  |  |
|  | Group GRH4 | Group T65 |  |  |  |  |
| Species | Av.Abund | Av.Abund | Av.Diss | Diss/SD | Contrib% | Cum.% |
| OTU_1 | 172.14 | 110.35 | 6.93 | 1.56 | 35.08 | 35.08 |
| OTU_17 | 87.84 | 56.01 | 3.44 | 2.69 | 17.41 | 52.49 |
| OTU_10 | 69.84 | 51.74 | 1.94 | 1.63 | 9.81 | 62.3 |
| OTU_12 | 63.08 | 46.22 | 1.86 | 1.48 | 9.4 | 71.7 |
| OTU_8 | 57.57 | 44.22 | 1.37 | 1.81 | 6.95 | 78.65 |

**Table S6** SIMPER analysis of leafhopper microbial communities (phase I) showing cumulative percentages of dissimilarity (based on average square-rooted abundance at the genus level) between significantly different pairs of the factor ‘natal’.

| Groups Batangas & Quezon | | |  |  |  |  |
| --- | --- | --- | --- | --- | --- | --- |
| Average dissimilarity = 26.75 | | |  |  |  |  |
|  |  |  |  |  |  |  |
|  | Group Batangas | Group Quezon |  |  |  |  |
| Species | Av.Abund | Av.Abund | Av.Diss | Diss/SD | Contrib% | Cum.% |
| *Candidatus sulcia* | 153.64 | 249.04 | 5.05 | 4.68 | 18.87 | 18.87 |
| *Cedecea* | 8.09 | 64.93 | 2.99 | 4.31 | 11.18 | 30.04 |
| *Kingella* | 77.12 | 130.32 | 2.79 | 1.92 | 10.41 | 40.45 |
| *Gluconobacter* | 63.26 | 114.12 | 2.68 | 3.65 | 10.02 | 50.47 |
| *Tenuibacillus* | 44.25 | 2.08 | 2.22 | 7.53 | 8.29 | 58.77 |
| *Staphylococcus* | 27.65 | 61.59 | 1.79 | 3.47 | 6.67 | 65.44 |
| *Diplorickettsia* | 36.23 | 32.96 | 1.4 | 1.09 | 5.24 | 70.68 |
| *Streptococcus* | 69.8 | 43.87 | 1.36 | 1.75 | 5.09 | 75.77 |
| Unclassified | 49.89 | 74.78 | 1.31 | 1.76 | 4.9 | 80.67 |
| *Neisseria* | 116.59 | 137.7 | 1.11 | 1.13 | 4.15 | 84.82 |
| *Piscinibacter* | 18.55 | 0.68 | 0.94 | 5.88 | 3.5 | 88.32 |
| *Pseudomonas* | 21.89 | 6.75 | 0.8 | 6.18 | 2.98 | 91.3 |
|  |  |  |  |  |  |  |
| Groups Laguna & Quezon | | |  |  |  |  |
| Average dissimilarity = 32.16 | | |  |  |  |  |
|  |  |  |  |  |  |  |
|  | Group Laguna | Group Quezon |  |  |  |  |
| Species | Av.Abund | Av.Abund | Av.Diss | Diss/SD | Contrib% | Cum.% |
| *Candidatus sulcia* | 152.29 | 249.04 | 5.24 | 3.48 | 16.29 | 16.29 |
| *Kingella* | 52.26 | 130.32 | 4.22 | 4.42 | 13.12 | 29.41 |
| *Gluconobacter* | 52.05 | 114.12 | 3.37 | 2.46 | 10.48 | 39.89 |
| *Cedecea* | 3.78 | 64.93 | 3.28 | 7.33 | 10.21 | 50.1 |
| *Tenuibacillus* | 46.55 | 2.08 | 2.41 | 2.92 | 7.5 | 57.6 |
| *Staphylococcus* | 16.96 | 61.59 | 2.4 | 11.18 | 7.47 | 65.07 |
| *Diplorickettsia* | 25.07 | 32.96 | 1.51 | 1.86 | 4.71 | 69.78 |
| *Streptococcus* | 70.09 | 43.87 | 1.45 | 0.94 | 4.5 | 74.27 |
| *Neisseria* | 114.65 | 137.7 | 1.42 | 1.7 | 4.42 | 78.7 |
| *Piscinibacter* | 26.41 | 0.68 | 1.39 | 3.24 | 4.33 | 83.03 |
| Unclassified | 70.99 | 74.78 | 1.07 | 1.41 | 3.31 | 86.34 |
| *Pseudomonas* | 22.02 | 6.75 | 0.83 | 2.36 | 2.58 | 88.92 |
| *Bradyrhizobium* | 33.31 | 41.18 | 0.81 | 2.09 | 2.51 | 91.43 |
|  |  |  |  |  |  |  |
| Groups Batangas & Rizal | | |  |  |  |  |
| Average dissimilarity = 29.99 | | |  |  |  |  |
|  |  |  |  |  |  |  |
|  | Group Batangas | Group Rizal |  |  |  |  |
| Species | Av.Abund | Av.Abund | Av.Diss | Diss/SD | Contrib% | Cum.% |
| *Candidatus sulcia* | 153.64 | 279.58 | 6.65 | 3.8 | 22.18 | 22.18 |
| *Cedecea* | 8.09 | 80.29 | 3.83 | 6.56 | 12.75 | 34.93 |
| Unclassified | 49.89 | 115.47 | 3.46 | 2.18 | 11.55 | 46.48 |
| *Gluconobacter* | 63.26 | 114.91 | 2.71 | 1.32 | 9.04 | 55.52 |
| *Neisseria* | 116.59 | 73.93 | 2.25 | 7.81 | 7.49 | 63.02 |
| *Tenuibacillus* | 44.25 | 2.59 | 2.21 | 5.1 | 7.38 | 70.4 |
| *Diplorickettsia* | 36.23 | 64 | 2.13 | 1.22 | 7.09 | 77.49 |
| *Bosea* | 43.26 | 69.11 | 1.36 | 3.02 | 4.54 | 82.03 |
| *Streptococcus* | 69.8 | 52.12 | 1.01 | 1.4 | 3.35 | 85.39 |
| *Piscinibacter* | 18.55 | 1.34 | 0.91 | 3.63 | 3.05 | 88.43 |
| *Bradyrhizobium* | 42.24 | 35.19 | 0.7 | 4.06 | 2.35 | 90.78 |
|  |  |  |  |  |  |  |
| Groups Laguna & Rizal | | |  |  |  |  |
| Average dissimilarity = 33.49 | | |  |  |  |  |
|  |  |  |  |  |  |  |
|  | Group Laguna | Group Rizal |  |  |  |  |
| Species | Av.Abund | Av.Abund | Av.Diss | Diss/SD | Contrib% | Cum.% |
| *Candidatus sulcia* | 152.29 | 279.58 | 6.88 | 3.13 | 20.53 | 20.53 |
| *Cedecea* | 3.78 | 80.29 | 4.14 | 9.42 | 12.36 | 32.89 |
| *Gluconobacter* | 52.05 | 114.91 | 3.8 | 1.69 | 11.35 | 44.24 |
| *Diplorickettsia* | 25.07 | 64 | 3.05 | 1.45 | 9.11 | 53.35 |
| Unclassified | 70.99 | 115.47 | 2.39 | 1.59 | 7.12 | 60.47 |
| *Tenuibacillus* | 46.55 | 2.59 | 2.37 | 3.87 | 7.07 | 67.54 |
| *Neisseria* | 114.65 | 73.93 | 2.19 | 4.09 | 6.55 | 74.1 |
| *Kingella* | 52.26 | 83.97 | 1.7 | 1.24 | 5.09 | 79.18 |
| *Piscinibacter* | 26.41 | 1.34 | 1.35 | 4.11 | 4.03 | 83.21 |
| *Streptococcus* | 70.09 | 52.12 | 0.97 | 0.82 | 2.88 | 86.1 |
| *Bradyrhizobium* | 33.31 | 35.19 | 0.92 | 2.06 | 2.75 | 88.85 |
| *Bosea* | 53.91 | 69.11 | 0.81 | 1.27 | 2.42 | 91.27 |
|  |  |  |  |  |  |  |
| Groups Quezon & Rizal | | |  |  |  |  |
| Average dissimilarity = 18.42 | | |  |  |  |  |
|  |  |  |  |  |  |  |
|  | Group Quezon | Group Rizal |  |  |  |  |
| Species | Av.Abund | Av.Abund | Av.Diss | Diss/SD | Contrib% | Cum.% |
| *Neisseria* | 137.7 | 73.93 | 2.97 | 4.53 | 16.15 | 16.15 |
| *Diplorickettsia* | 32.96 | 64 | 2.9 | 1.19 | 15.75 | 31.9 |
| *Kingella* | 130.32 | 83.97 | 2.17 | 2.43 | 11.76 | 43.66 |
| Unclassified | 74.78 | 115.47 | 1.89 | 1.9 | 10.27 | 53.93 |
| *Staphylococcus* | 61.59 | 27.67 | 1.58 | 5.32 | 8.59 | 62.51 |
| *Candidatus Sulcia* | 249.04 | 279.58 | 1.44 | 1.1 | 7.81 | 70.32 |
| *Gluconobacter* | 114.12 | 114.91 | 1.14 | 1.05 | 6.19 | 76.51 |
| *Bosea* | 47.81 | 69.11 | 0.99 | 3.36 | 5.38 | 81.89 |
| *Cedecea* | 64.93 | 80.29 | 0.72 | 1.55 | 3.9 | 85.79 |
| *Bradyrhizobium* | 41.18 | 35.19 | 0.49 | 1.67 | 2.65 | 88.44 |
| *Streptococcus* | 43.87 | 52.12 | 0.49 | 2.12 | 2.65 | 91.08 |
|  |  |  |  |  |  |  |
| Groups Batangas & SanPablo | | |  |  |  |  |
| Average dissimilarity = 36.51 | | |  |  |  |  |
|  |  |  |  |  |  |  |
|  | Group Batangas | Group SanPablo |  |  |  |  |
| Species | Av.Abund | Av.Abund | Av.Diss | Diss/SD | Contrib% | Cum.% |
| *Paenirhodobacter* | 2.55 | 170.86 | 9.31 | 3.97 | 25.51 | 25.51 |
| Unclassified | 49.89 | 167.21 | 6.35 | 8.18 | 17.4 | 42.91 |
| *Streptococcus* | 69.8 | 6.67 | 3.41 | 5.28 | 9.35 | 52.25 |
| *Tenuibacillus* | 44.25 | 2.99 | 2.25 | 7.46 | 6.15 | 58.4 |
| *Bosea* | 43.26 | 79.07 | 1.92 | 2.47 | 5.25 | 63.66 |
| *Kingella* | 77.12 | 62.48 | 1.74 | 1.73 | 4.77 | 68.43 |
| *Neisseria* | 116.59 | 117.2 | 1.61 | 1.15 | 4.42 | 72.85 |
| *Bradyrhizobium* | 42.24 | 63.94 | 1.55 | 1.61 | 4.24 | 77.09 |
| *Candidatus sulcia* | 153.64 | 182.01 | 1.53 | 0.85 | 4.19 | 81.28 |
| *Gluconobacter* | 63.26 | 42.89 | 1.46 | 1.54 | 4 | 85.28 |
| *Diplorickettsia* | 36.23 | 28.33 | 1.11 | 1.76 | 3.04 | 88.31 |
| *Piscinibacter* | 18.55 | 1.35 | 0.94 | 6.54 | 2.56 | 90.88 |
|  |  |  |  |  |  |  |
| Groups Laguna & SanPablo | | |  |  |  |  |
| Average dissimilarity = 37.44 | | |  |  |  |  |
|  |  |  |  |  |  |  |
|  | Group Laguna | Group SanPablo |  |  |  |  |
| Species | Av.Abund | Av.Abund | Av.Diss | Diss/SD | Contrib% | Cum.% |
| *Paenirhodobacter* | 3.85 | 170.86 | 9.39 | 4.55 | 25.07 | 25.07 |
| Unclassified | 70.99 | 167.21 | 5.31 | 2.77 | 14.17 | 39.24 |
| *Streptococcus* | 70.09 | 6.67 | 3.53 | 2.86 | 9.42 | 48.66 |
| *Tenuibacillus* | 46.55 | 2.99 | 2.43 | 3.11 | 6.5 | 55.16 |
| *Bradyrhizobium* | 33.31 | 63.94 | 2.35 | 3.67 | 6.29 | 61.45 |
| *Neisseria* | 114.65 | 117.2 | 1.89 | 2.17 | 5.04 | 66.49 |
| *Candidatus Sulcia* | 152.29 | 182.01 | 1.82 | 0.75 | 4.85 | 71.33 |
| *Kingella* | 52.26 | 62.48 | 1.6 | 1.53 | 4.26 | 75.6 |
| *Gluconobacter* | 52.05 | 42.89 | 1.43 | 1.13 | 3.81 | 79.41 |
| *Piscinibacter* | 26.41 | 1.35 | 1.41 | 3.18 | 3.76 | 83.17 |
| *Bosea* | 53.91 | 79.07 | 1.38 | 1.18 | 3.68 | 86.85 |
| *Diplorickettsia* | 25.07 | 28.33 | 1.12 | 1.11 | 3 | 89.84 |
| Staphylococcus | 16.96 | 35.59 | 1.02 | 2.43 | 2.72 | 92.57 |
|  |  |  |  |  |  |  |
| Groups Quezon & SanPablo | | |  |  |  |  |
| Average dissimilarity = 35.76 | | |  |  |  |  |
|  |  |  |  |  |  |  |
|  | Group Quezon | Group SanPablo |  |  |  |  |
| Species | Av.Abund | Av.Abund | Av.Diss | Diss/SD | Contrib% | Cum.% |
| *Paenirhodobacter* | 1.58 | 170.86 | 8.18 | 4.3 | 22.86 | 22.86 |
| Unclassified | 74.78 | 167.21 | 4.34 | 3.74 | 12.14 | 35 |
| *Gluconobacter* | 114.12 | 42.89 | 3.46 | 3.04 | 9.68 | 44.69 |
| *Kingella* | 130.32 | 62.48 | 3.29 | 2.77 | 9.2 | 53.89 |
| *Candidatus Sulcia* | 249.04 | 182.01 | 3.25 | 2.2 | 9.1 | 62.98 |
| *Cedecea* | 64.93 | 3.77 | 2.96 | 3.31 | 8.29 | 71.27 |
| *Streptococcus* | 43.87 | 6.67 | 1.78 | 7.95 | 4.98 | 76.25 |
| *Bosea* | 47.81 | 79.07 | 1.46 | 1.6 | 4.08 | 80.33 |
| *Bradyrhizobium* | 41.18 | 63.94 | 1.3 | 2.23 | 3.63 | 83.95 |
| *Staphylococcus* | 61.59 | 35.59 | 1.27 | 2.44 | 3.55 | 87.5 |
| *Diplorickettsia* | 32.96 | 28.33 | 1.27 | 2.19 | 3.55 | 91.05 |
|  |  |  |  |  |  |  |
| Groups Rizal & SanPablo | | |  |  |  |  |
| Average dissimilarity = 35.15 | | |  |  |  |  |
|  |  |  |  |  |  |  |
|  | Group Rizal | Group SanPablo |  |  |  |  |
| Species | Av.Abund | Av.Abund | Av.Diss | Diss/SD | Contrib% | Cum.% |
| *Paenirhodobacter* | 2.29 | 170.86 | 8.16 | 4.58 | 23.21 | 23.21 |
| *Candidatus sulcia* | 279.58 | 182.01 | 4.71 | 4.02 | 13.39 | 36.6 |
| *Cedecea* | 80.29 | 3.77 | 3.71 | 3.88 | 10.56 | 47.15 |
| *Gluconobacter* | 114.91 | 42.89 | 3.42 | 3.89 | 9.72 | 56.88 |
| Unclassified | 115.47 | 167.21 | 2.84 | 1.7 | 8.08 | 64.95 |
| *Diplorickettsia* | 64 | 28.33 | 2.28 | 1.02 | 6.49 | 71.45 |
| *Streptococcus* | 52.12 | 6.67 | 2.19 | 6.36 | 6.23 | 77.67 |
| *Neisseria* | 73.93 | 117.2 | 2.18 | 1.39 | 6.19 | 83.87 |
| *Bradyrhizobium* | 35.19 | 63.94 | 1.36 | 1.18 | 3.87 | 87.73 |
| *Kingella* | 83.97 | 62.48 | 1.28 | 1.36 | 3.65 | 91.39 |

**Table S7** SIMPER analysis of leafhopper microbial communities (phase II) showing cumulative percentages of dissimilarity (based on average square-rooted abundance at the OTU level) between significantly different pairs of the factor ‘location’.

|  |  |  |  |  |  |  |
| --- | --- | --- | --- | --- | --- | --- |
| Groups Batangas & Laguna | | |  |  |  |  |
| Average dissimilarity = 31.55 | | |  |  |  |  |
|  |  |  |  |  |  |  |
|  | Group Batangas | Group Laguna |  |  |  |  |
| Species | Av.Abund | Av.Abund | Av.Diss | Diss/SD | Contrib% | Cum.% |
| OTU_17 | 59.76 | 124.05 | 6.86 | 2.04 | 21.75 | 21.75 |
| OTU_1 | 138.3 | 196.9 | 6.53 | 2.16 | 20.7 | 42.45 |
| OTU_8 | 43.95 | 99.37 | 5.81 | 2.08 | 18.41 | 60.86 |
| OTU_12 | 54.83 | 100.66 | 4.88 | 1.93 | 15.45 | 76.32 |
| OTU_10 | 52.43 | 83.15 | 3.33 | 1.74 | 10.56 | 86.87 |
|  |  |  |  |  |  |  |
|  |  |  |  |  |  |  |
| Groups Batangas & SanPablo | | |  |  |  |  |
| Average dissimilarity = 28.79 | | |  |  |  |  |
|  |  |  |  |  |  |  |
|  | Group Batangas | Group SanPablo |  |  |  |  |
| Species | Av.Abund | Av.Abund | Av.Diss | Diss/SD | Contrib% | Cum.% |
| OTU_1 | 138.3 | 235.23 | 10.26 | 2.12 | 35.63 | 35.63 |
| OTU_17 | 59.76 | 96.5 | 5.38 | 3.09 | 18.68 | 54.31 |
| OTU_10 | 52.43 | 85.71 | 3.74 | 1.7 | 13 | 67.31 |
| OTU_12 | 54.83 | 70.74 | 2.92 | 1.83 | 10.13 | 77.44 |
| OTU_8 | 43.95 | 54.58 | 2.89 | 2.35 | 10.03 | 87.47 |
|  |  |  |  |  |  |  |
| Groups Laguna & SanPablo | | |  |  |  |  |
| Average dissimilarity = 14.79 | | |  |  |  |  |
|  |  |  |  |  |  |  |
|  | Group Laguna | Group SanPablo |  |  |  |  |
| Species | Av.Abund | Av.Abund | Av.Diss | Diss/SD | Contrib% | Cum.% |
| OTU_8 | 99.37 | 54.58 | 3.39 | 1.85 | 22.91 | 22.91 |
| OTU_1 | 196.9 | 235.23 | 3.1 | 1.75 | 20.98 | 43.89 |
| OTU_12 | 100.66 | 70.74 | 2.44 | 1.38 | 16.48 | 60.37 |
| OTU_17 | 124.05 | 96.5 | 2.21 | 1.49 | 14.97 | 75.34 |
| OTU_10 | 83.15 | 85.71 | 1.56 | 1.37 | 10.54 | 85.88 |
|  |  |  |  |  |  |  |
| Groups Quezon & SanPablo | | |  |  |  |  |
| Average dissimilarity = 14.25 | | |  |  |  |  |
|  |  |  |  |  |  |  |
|  | Group Quezon | Group SanPablo |  |  |  |  |
| Species | Av.Abund | Av.Abund | Av.Diss | Diss/SD | Contrib% | Cum.% |
| OTU_1 | 186.72 | 235.23 | 4.16 | 1.49 | 29.19 | 29.19 |
| OTU_8 | 90.45 | 54.58 | 2.81 | 2.03 | 19.72 | 48.91 |
| OTU_17 | 116.78 | 96.5 | 1.97 | 1.55 | 13.85 | 62.76 |
| OTU_12 | 87.89 | 70.74 | 1.7 | 1.41 | 11.93 | 74.69 |
| OTU_10 | 84.2 | 85.71 | 1.42 | 1.37 | 9.96 | 84.65 |
|  |  |  |  |  |  |  |
| Groups Rizal & SanPablo | | |  |  |  |  |
| Average dissimilarity = 16.81 | | |  |  |  |  |
|  |  |  |  |  |  |  |
|  | Group Rizal | Group SanPablo |  |  |  |  |
| Species | Av.Abund | Av.Abund | Av.Diss | Diss/SD | Contrib% | Cum.% |
| OTU_1 | 164.13 | 235.23 | 6.25 | 1.68 | 37.19 | 37.19 |
| OTU_8 | 91.3 | 54.58 | 3 | 2.06 | 17.88 | 55.07 |
| OTU_17 | 99.23 | 96.5 | 2.01 | 1.36 | 11.98 | 67.05 |
| OTU_12 | 87.34 | 70.74 | 1.77 | 1.44 | 10.56 | 77.6 |
| OTU_10 | 91.39 | 85.71 | 1.62 | 1.37 | 9.66 | 87.27 |

**Table S8** SIMPER analysis of leafhopper microbial communities (phase II) showing cumulative percentages of dissimilarity (based on average square-rooted abundance at the genus level) between significantly different pairs of locations.

| Groups Batangas & Laguna | |  |  |  |  |  |
| --- | --- | --- | --- | --- | --- | --- |
| Average dissimilarity = 37.20 | |  |  |  |  |  |
|  |  |  |  |  |  |  |
|  | Group Batangas | Group Laguna |  |  |  |  |
| Species | Av.Abund | Av.Abund | Av.Diss | Diss/SD | Contrib% | Cum.% |
| Unclassified | 195.42 | 53.66 | 7.76 | 2.06 | 20.85 | 20.85 |
| *Paenirhodobacter* | 123.01 | 1.08 | 6.7 | 1.85 | 18 | 38.85 |
| *Candidatus sulcia* | 178.2 | 290.21 | 6.66 | 2.25 | 17.9 | 56.74 |
| *Gluconobacter* | 70.22 | 126.14 | 3.29 | 1.49 | 8.86 | 65.6 |
| *Streptococcus* | 14.77 | 58.7 | 2.79 | 1.63 | 7.49 | 73.09 |
| *Kingella* | 47.72 | 76.01 | 1.93 | 1.41 | 5.18 | 78.27 |
| *Neisseria* | 67.21 | 74.96 | 1.84 | 4.57 | 4.94 | 83.21 |
| *Bradyrhizobium* | 49.35 | 29.36 | 1.17 | 1.28 | 3.14 | 86.35 |
| *Staphylococcus* | 35.7 | 38.95 | 1.04 | 2.2 | 2.79 | 89.14 |
| *Bosea* | 80.76 | 78.54 | 0.95 | 1.44 | 2.56 | 91.7 |
|  |  |  |  |  |  |  |
| Groups Batangas & Quezon | |  |  |  |  |  |
| Average dissimilarity = 34.58 | |  |  |  |  |  |
|  |  |  |  |  |  |  |
|  | Group Batangas | Group Quezon |  |  |  |  |
| Species | Av.Abund | Av.Abund | Av.Diss | Diss/SD | Contrib% | Cum.% |
| *Paenirhodobacter* | 123.01 | 0.68 | 6.91 | 1.85 | 19.99 | 19.99 |
| Unclassified | 195.42 | 83.72 | 6.72 | 2.11 | 19.42 | 39.42 |
| *Candidatus sulcia* | 178.2 | 271.2 | 6.18 | 2.2 | 17.88 | 57.3 |
| *Gluconobacter* | 70.22 | 128.34 | 3.64 | 1.49 | 10.53 | 67.83 |
| *Streptococcus* | 14.77 | 53.42 | 2.76 | 1.15 | 7.98 | 75.81 |
| *Kingella* | 47.72 | 69.85 | 1.64 | 2.26 | 4.74 | 80.55 |
| *Bradyrhizobium* | 49.35 | 24.41 | 1.41 | 1.58 | 4.07 | 84.62 |
| *Bosea* | 80.76 | 59.9 | 1.32 | 1.57 | 3.82 | 88.44 |
| *Neisseria* | 67.21 | 56.68 | 0.7 | 1.75 | 2.02 | 90.47 |
|  |  |  |  |  |  |  |
| Groups Batangas & SanPablo | |  |  |  |  |  |
| Average dissimilarity = 38.30 | |  |  |  |  |  |
|  |  |  |  |  |  |  |
|  | Group Batangas | Group SanPablo |  |  |  |  |
| Species | Av.Abund | Av.Abund | Av.Diss | Diss/SD | Contrib% | Cum.% |
| Unclassified | 195.42 | 44.28 | 8.27 | 2.14 | 21.59 | 21.59 |
| *Paenirhodobacter* | 123.01 | 0.45 | 6.69 | 1.84 | 17.48 | 39.07 |
| *Candidatus sulcia* | 178.2 | 285.95 | 6.51 | 2.02 | 16.99 | 56.06 |
| *Gluconobacter* | 70.22 | 116.9 | 3.54 | 1.36 | 9.24 | 65.31 |
| *Diplorickettsia* | 16.32 | 49.01 | 2.26 | 1.16 | 5.89 | 71.2 |
| *Cedecea* | 5.56 | 42.53 | 1.99 | 2 | 5.19 | 76.38 |
| *Neisseria* | 67.21 | 87.08 | 1.84 | 1.64 | 4.81 | 81.19 |
| *Kingella* | 47.72 | 66.18 | 1.76 | 1.86 | 4.6 | 85.79 |
| *Bosea* | 80.76 | 86.76 | 0.99 | 1.3 | 2.58 | 88.36 |
| *Bradyrhizobium* | 49.35 | 47.47 | 0.91 | 1.16 | 2.37 | 90.74 |
|  |  |  |  |  |  |  |
| Groups Laguna & SanPablo | |  |  |  |  |  |
| Average dissimilarity = 22.04 | |  |  |  |  |  |
|  |  |  |  |  |  |  |
|  | Group Laguna | Group SanPablo |  |  |  |  |
| Species | Av.Abund | Av.Abund | Av.Diss | Diss/SD | Contrib% | Cum.% |
| *Streptococcus* | 58.7 | 1.17 | 3.19 | 1.83 | 14.49 | 14.49 |
| *Gluconobacter* | 126.14 | 116.9 | 2.88 | 1.95 | 13.06 | 27.55 |
| *Diplorickettsia* | 13.73 | 49.01 | 2.41 | 1.18 | 10.96 | 38.51 |
| *Neisseria* | 74.96 | 87.08 | 2.17 | 1.28 | 9.83 | 48.34 |
| *Cedecea* | 5.91 | 42.53 | 2 | 1.9 | 9.08 | 57.42 |
| *Kingella* | 76.01 | 66.18 | 1.72 | 1.46 | 7.81 | 65.23 |
| *Candidatus sulcia* | 290.21 | 285.95 | 1.39 | 1.2 | 6.32 | 71.55 |
| *Staphylococcus* | 38.95 | 24.38 | 1.2 | 1.53 | 5.46 | 77.01 |
| *Bosea* | 78.54 | 86.76 | 1.16 | 1.39 | 5.27 | 82.28 |
| Unclassified | 53.66 | 44.28 | 1.1 | 1.56 | 5 | 87.27 |
| *Bradyrhizobium* | 29.36 | 47.47 | 1.03 | 1.03 | 4.68 | 91.95 |
|  |  |  |  |  |  |  |
| Groups Quezon & SanPablo | |  |  |  |  |  |
| Average dissimilarity = 23.35 | |  |  |  |  |  |
|  |  |  |  |  |  |  |
|  | Group Quezon | Group SanPablo |  |  |  |  |
| Species | Av.Abund | Av.Abund | Av.Diss | Diss/SD | Contrib% | Cum.% |
| *Gluconobacter* | 128.34 | 116.9 | 3.05 | 1.67 | 13.04 | 13.04 |
| *Streptococcus* | 53.42 | 1.17 | 2.95 | 1.12 | 12.65 | 25.7 |
| *Diplorickettsia* | 15.54 | 49.01 | 2.42 | 1.15 | 10.37 | 36.06 |
| Unclassified | 83.72 | 44.28 | 2.25 | 1.77 | 9.65 | 45.72 |
| *Neisseria* | 56.68 | 87.08 | 2.16 | 1.53 | 9.27 | 54.99 |
| *Candidatus sulcia* | 271.2 | 285.95 | 2.11 | 1.16 | 9.05 | 64.03 |
| *Cedecea* | 7.56 | 42.53 | 1.97 | 1.86 | 8.45 | 72.49 |
| *Bosea* | 59.9 | 86.76 | 1.67 | 1.45 | 7.17 | 79.65 |
| *Bradyrhizobium* | 24.41 | 47.47 | 1.27 | 1.31 | 5.44 | 85.1 |
| *Kingella* | 69.85 | 66.18 | 1.21 | 1.22 | 5.2 | 90.3 |
|  |  |  |  |  |  |  |
| Groups Rizal & SanPablo | |  |  |  |  |  |
| Average dissimilarity = 25.83 | |  |  |  |  |  |
|  |  |  |  |  |  |  |
|  | Group Rizal | Group SanPablo |  |  |  |  |
| Species | Av.Abund | Av.Abund | Av.Diss | Diss/SD | Contrib% | Cum.% |
| Unclassified | 119.21 | 44.28 | 4.26 | 1.65 | 16.48 | 16.48 |
| *Candidatus sulcia* | 251.99 | 285.95 | 2.95 | 1.32 | 11.4 | 27.88 |
| *Gluconobacter* | 117.13 | 116.9 | 2.91 | 1.37 | 11.28 | 39.16 |
| *Diplorickettsia* | 20.59 | 49.01 | 2.27 | 1.19 | 8.79 | 47.94 |
| *Neisseria* | 63.73 | 87.08 | 1.97 | 1.57 | 7.61 | 55.55 |
| *Kingella* | 94.57 | 66.18 | 1.9 | 1.26 | 7.34 | 62.9 |
| *Cedecea* | 9.83 | 42.53 | 1.85 | 1.77 | 7.17 | 70.06 |
| *Bosea* | 54.64 | 86.76 | 1.83 | 1.53 | 7.08 | 77.14 |
| *Streptococcus* | 33.57 | 1.17 | 1.82 | 2.38 | 7.05 | 84.19 |
| *Bradyrhizobium* | 25.13 | 47.47 | 1.62 | 1.91 | 6.27 | 90.46 |
